# Controlled synthesis of graphene oxide/silica hybrid nanocomposites for removal of aromatic pollutants in water

**DOI:** 10.1038/s41598-022-10602-4

**Published:** 2022-04-29

**Authors:** Amr Abdelkhalek, Mona Abd El-Latif, Hesham Ibrahim, Hesham Hamad, Marwa Showman

**Affiliations:** 1 Department of Environmental Studies, Institute of Graduate Studies and Research (IGSR), Alexandria University, P.O. Box 832, Alexandria, Egypt; 2 Fabrication Technology Research Department, Advanced Technology and New Materials Research Institute (ATNMRI), City of Scientific Research and Technological Applications (SRTA-City), New Borg El-Arab City, Alexandria, 21934 Egypt

**Keywords:** Materials science, Environmental chemistry, Materials chemistry, Physical chemistry, Chemical synthesis

## Abstract

The remarkable characteristics of graphene make it a model candidate for boosting the effectiveness of nano-adsorbents with high potential owing to its large surface area, π–π interaction, and accessible functional groups that interact with an adsorbate. However, the stacking of graphene reduces its influence adsorption characteristics and also its practical application. On the other hand, the widespread use of aromatic compounds in the industry has aggravated the contamination of the water environment, and how to effectively remove them has become a research hotspot. Herein, we develop the functionalization of silica nanoparticles on graphene oxide nanosheet (FGS) by a facile, cheap, and efficient synthesis protocol for adsorption of Trypan Blue (TB) and Bisphenol A (BPA). It was demonstrated that chemical activation with KOH at high autoclaving temperature successfully transformed rice husk ash (RHA) into FGS. The graphene oxide layered interlamination was kept open by using SiO_2_ to expose the interlayers' strong adsorption sites. XRD, EDX, FTIR, Raman spectroscopy, SEM, HR-TEM, and BET surface area are used to investigate the chemical composition, structure, morphology, and textural nature of the as-produced FGS hybrid nanocomposite. The various oxygen-containing functional groups of the hybrid nanocomposites resulted in a significantly increased adsorption capacity, according to experimental findings. In addition, FGS2, the best composite, has a specific surface area of 1768 m^2^g^−1^. Based on Langmuir isotherms, the maximal TB dye and BPA removal capacity attained after 30 min were 455 and 500 mg/g, respectively. The Langmuir isotherm model, a pseudo-second-order kinetic model, and an intraparticle diffusion model have all been used to provide mechanistic insights into the adsorption process. This suggests that BPA and TB adsorption on FGS2 is mostly chemically regulated monolayer adsorption. Due to its unique sp^2^-hybridized single-atom-layer structure, the exposed graphene oxide nanosheets' extremely hydrophobic effect, hydrogen bonding, and strong—electron donor–acceptor interaction contributed to their improved adsorption of BPA and TB. According to adsorption thermodynamics, FGS2 adsorption of TB and BPA is a spontaneous exothermic reaction that is aided by lowering the temperature. For adsorption-based wastewater cleanup, the produced nanocomposites with a regulated amount of carbon and silica in the form of graphene oxide and silica can be used. These findings suggest that functionalized GO/SiO_2_ hybrid nanocomposites could be a viable sorbent for the efficient and cost-effective removal of aromatic chemicals from wastewater.

## Introduction

Providing safe and clean water in the face of scarcity and pollution is one of the most pressing global challenges of the twenty-first century. The scarcity of safe drinking water is a major worry for all living species on the planet, not just humans. As a result, one of humanity's greatest challenges in the modern era is the scarcity of pure, uncontaminated freshwater. The industrial sector is the primary generator of pollution and has a direct detrimental influence on the environment in all aspects. Because most organic contaminants are toxic and carcinogenic and slow to degrade, wastewater treatment technology that is both environmentally friendly and financially effective is continuously in demand^1^.

Synthetic organic chemicals including dyes and phenols, as well as their derivatives, have been discovered as common anthropogenic contaminants in industrial effluent. Because they are virtually carcinogenic or mutagenic agents with high toxicity in acute amounts, they have the same characteristics in any industrial effluent contaminated with them. The largest group of synthetic organic dyes is having a delocalized electron system are azo dyes, which are often persistent compounds with vivid hues because they absorb light in the visible area^2^. Trypan blue (TB) is a diazo dye with a complex structure and excellent chemical and photostability. It has been identified as a carcinogenic agent with the same dangers to human health and the environment as azo dyes^3^. In addition, phenolic chemicals released in industrial effluents have direct and negative consequences on the aquatic environment and human health. Phenols are poisonous and carcinogenic in general, and even at very low quantities of a microgram per liter, they can alter the taste and odor of drinking water^4^. Bisphenol A (BPA) is a phenol-containing chemical substance. It has become one of the most commonly made and consumed substances on the planet in recent decades. Because of the large scale of production and usage, BPA contamination is found in all environmental components, particularly the hydrosphere^5^. BPA exposure at high doses, according to convincing evidence, can cause carcinogenic and mutagenic changes. BPA also has deleterious effects on male reproduction, which is linked to metabolic syndrome, as well as thyroid gland function, hypertension, and cardiovascular disease^5,6^.

Both TB and BPA are emerging synthetic pollutants rapidly diffused in the aquatic environment. The conventional technologies for removing dyes and phenols from wastewater are including photocatalytic degradation^7^, nanofiltration^8^, anodic oxidation^9^, electrocoagulation^10^, and biological treatment^11^. Because of its efficiency, cost, and ease of operation, adsorption technology is an excellent choice for removal of organic pollutants^12^.

Carbon allotropes are classified into four classes based on their geometry: 0-D, which includes fullerenes^13^, 1-D, which includes carbon nanotubes (CNTs)^14^, 2-D, which includes graphene and graphene oxides^15^, and 3-D, which includes activated carbon (AC)^16^. All of these kinds of carbon allotropes have previously been shown to be good adsorbents for the elimination of organic contaminants^13–16^. Due to its high specific surface area, excellent chemical stability, low cost of mass production, simple operation, and easy regeneration, graphene and its derivatives, as well as activated carbon, are the most extensively utilized adsorbents for water treatment^17^. In addition, graphene and its derivatives have a large surface area, superior electronic and mechanical properties, exceptional charge transport mobility, and very high thermal conductivity, suggesting that they could be used in a variety of applications including solar cells, supercapacitors, sensors, solar cells, and adsorbents^18^. The ability of graphene to selectively remove aromatic compounds defined by benzene rings via a strong bond-interaction is its primary advantage over other carbonaceous materials. This material also has the advantage of being manufactured from low-cost graphite found in agricultural waste^19^. Furthermore, graphene has a strong ability to interact with other materials, such as silica, to form novel nanocomposites with improved removal properties.

As a result, developing new carbonaceous adsorbents, such as graphene nanocomposites, from low-cost natural precursors with high adsorptive capacity, high adsorption rate, and a large surface area for reactivity, is crucial. Several efforts are presently underway to develop green synthesis methods for graphene production. The purpose of green synthesis methods is to use natural precursors to substitute hazardous chemicals^15^.

On the other hands, to solve the problem of stacking and aggregation of graphene layers by providing a space layer of inorganic nanomaterials like silica nanaoparticles as well as it has excellent adsorption characteristics^20^. As a result, the hybridization of nanosilica with graphene is targeted to explore new characteristics. Silica could disperse densely and uniformly on the GO surface, providing GO/ SiO_2_ nanocomposites a high hydrophilicity^21^. Some studies have reported on the characteristics of GO/SiO_2_ composites, implying that they are suitable for use in the removal of large amounts of contaminants by adsorption or membranes^22,23^.

Low-cost adsorbents made from solid agricultural wastes have received a lot of attention in recent decades^24,25^. Agricultural wastes like rice husk (RH) are an efficient byproduct that is commonly employed as biomass in energy generation around the world^25^. Because of its local availability, low cost, granular structure, chemical stability, insolubility in water, and great mechanical strength, RH was chosen as a starting material. On the other hands, in Egypt, open field burning and landfilling are the most common disposal methods, which often result in greenhouse gas emissions, air pollution, and a large landfill space occupation. To solve those problems, trying to explore economic methods to make full use of rice husk instead of throwing it away is a necessity. In addition, methods to derive higher values from RH have been explored, such as various silicon-based materials, which have attracted high attention in the past decades. Harvesting silica and silica composites from RH can not only take full advantage of their highest potential value, but also minimize the related environmental issues from the valorization of RH^26^. Despite its high silica content, it has limited commercial value. As a result, developing stable nanocomposites made of rice husk ash (RHA) that can be employed as adsorbents to remove organic pollutants from industrial effluents is a crucial goal.

According to our knowledge, the novelty of this work is to gain better insights for the investigation of new series of GO/SiO_2_ nanocomposites from natural rice husk which are sustainable, abundant, and low-cost agricultural biomass as an adsorbent for removal of various organics. As a result, it can be replaced the expensive and unavailable materials for the preparation of GO in previous studies with locally available materials in large quantities in Egypt which is more effective in production on large scale for industrial applications. Another advantage of this research is the one-pot step approach for the synthesis of GO/SiO_2_ because it utilizes few chemicals and uses mild processing conditions. This cutting-edge material will be tested as a promising adsorbent for removing TB and BPA from aqueous solutions onto the produced GO/SiO_2_. In addition, kinetic, isotherm, and thermodynamic models will be used to suggest a thorough mechanism for the adsorption process onto GO/SiO_2_. This research benefits from the wastes-treat-wastes approach, which combines waste and water treatment, and adds to sustainable materials processing.

## Experimental procedures

### Starting materials and reagents

Rice husk (RH) was obtained from the local market that collected from the farmlands near to Alexandria, Egypt. Potassium hydroxide (KOH, 85%) was supplied from ADWIC, El-Nasr Company, Egypt. Trypan blue (TB) (70%) was purchased from Sigma Aldrich, Germany. Bisphenol A (97%) was supplied from ACROS, Belgium. All solutions were prepared with distilled water under ambient conditions.

### Synthesis of functionalized GO/SiO_2_ nanocomposites

The fresh RH was used as a cheap precursor for carbon and silica owing to its abundance. It was sieved to obtain uniform size, washed several times with distilled water to remove any contaminants, and dried at 105 °C in oven overnight.

**Figure 1:**
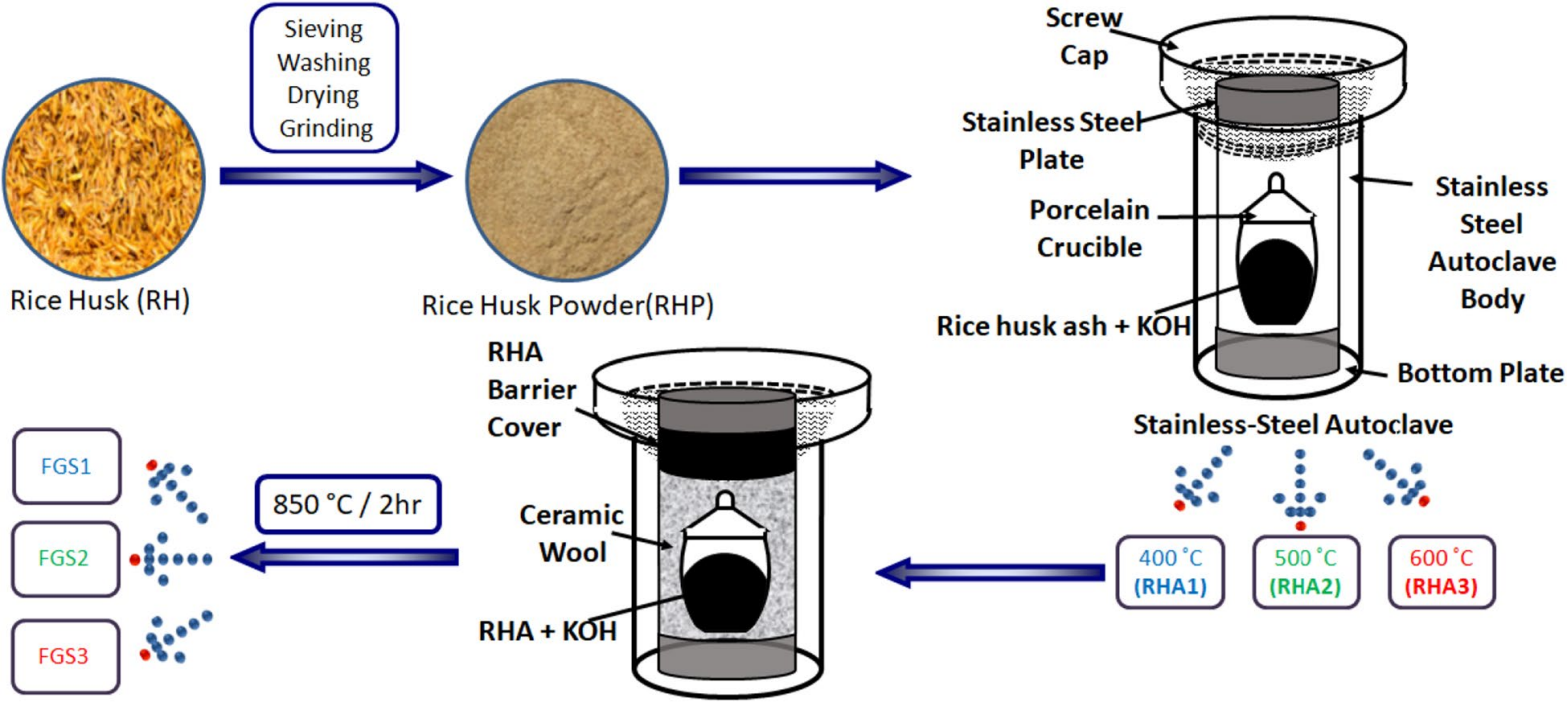
Schematic representation set up system for the synthesis of GO/SiO_2_ nanocomposites.

Functionalized graphene oxide (GO)/SiO_2_ nanocomposites (FGS) was successfully fabricated by two steps; the first one was the combustion of RHP in covered crucible inside stainless steel autoclave at three different temperature, 400, 500, and 600 °C at 1 h for obtaining ash RHA1, RHA2, and RHA3, respectively. The second step was the utilization of three samples as a precursor for the transformation of RHA into GO/SiO_2_ nanocomposites by the chemical activation with KOH at high autoclaving temperature. 3 g of RHA (RHA1, RHA2, RHA3) and 15 g KOH were mixed together by grinding in ceramic mortar for 15 min. The mixture compacted in a porcelain crucible, covered with ceramic wool, and fixed into stainless steel autoclave then activated at 850 °C for 2 h in a muffle furnace. After the activation treatment, the samples were washed with distilled water several times to remove the excess KOH, and then dried at 100 °C for 24 h. Three final products were obtained and entitled as FGS1, FGS2 and FGS3, respectively. Schematic setup system of the synthesis of GO/SiO_2_ nanocomposites is illustrated in Fig. 1. All prepared samples were monitored towards the adsorption of Trypan blue (TB) and Bisphenol A (BPA).

### Characterization of adsorbents, adsorption studies, kinetics, isotherms and thermodynamic adsorption behavior

The detailed characterization, and adsorption studies, kinetics, isotherms, and thermodynamic studies are stated in supporting information (S1-S3). The fundamental information for adjusting the experimental data to the mathematical kinetic and isotherm models was presented in Table S1.

## Result and discussion

Rice husk is an inexpensive source of functionalized nanomaterials that can be employed in a wide range of applications. As a result, one of the objectives is to make highly efficient and low-cost graphitic materials in a simple method. Chemical activation of rice husk ash as a precursor of carbon and SiO_2_ in the composite yielded a facile, efficient, and scalable technique for manufacturing functionalized graphene oxide with silica (FGS) in the current work. As a catalyst for RHA activation, KOH is a strong alkali.

### Characteristics of RHA

Rice husk chemical composition varies by harvest year, paddy type, and meteorological and geographic factors. Rice husk is high in silica and contains cellulose, hemicellulose, lignin, and ash, as well as other carbon compounds^25^. The clean dry husk was grinded in the mill to obtain powder with homogenous particle size of 355.8 nm (Fig. S1a) and after obtaining ash at 500 °C is 182.5 nm (Fig. S1b).

**Figure 2:**
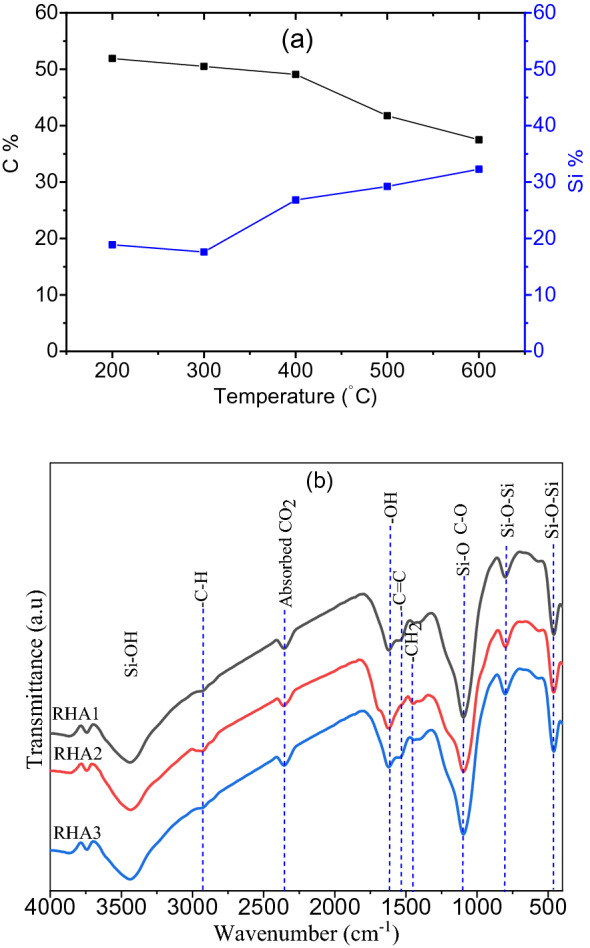
(**a**) Variation the percentage of C and Si with temperature, and (**b**) FTIR of RHA1, RHA2, and RHA3.

Figure 2a depicts the effect of temperature on the structure of the RHA generated. RHA's chemical composition was primarily carbon and silicon, with the carbon content being higher than the silicon percentage, according to EDX. When the temperature is increased from 200 to 600 °C, the C content declines while the Si content increases while the O content remains constant, indicating that increasing the oxidation state leads to the creation of SiO_2_. The adsorption qualities improve as the proportion of silica increases, as proven by FTIR.

FTIR spectra were used to identify the surface functional groups of the generated rice husk ash, RHA1, RHA2, and RHA3, as shown in Fig. 2b. A large absorption band in the region 3742–3867 cm^−1^ suggests the generation of free and hydrogen bound hydroxyl groups in physically adsorbed water and Si–OH, indicating the presence of free surface –OH stretching vibration^27^. The presence of C–H asymmetric stretching vibration, which attributes the carbon in the samples, is associated with the band at 2916 cm^−1^. The asymmetric stretching vibration of CO_2_ molecule corresponds to the peak at 2346 cm^−1^^28^. The peaks around 1615 cm^−1^ correspond to –OH bending vibration of adsorbed water and stretching vibration ascribed to the aromatic carboxyl groups of a conjugated carbonyl group (C=O and COO) in hemicelluloses and lignin^20^. The band at 1544 cm^−1^ is assigned to the C=C stretching vibration in polysaccharide rings^29^. In addition, the absorption band was observed at 1450 cm^−1^, due to the –CH_2_ bending. The absorption band at 1098 cm^−1^ attributed to the symmetrical stretching vibration mode of Si–O bond network consisting of Si–O–Si or O–Si–O^30^. On the other hands, in the same region at the strong band C–O groups in the polysaccharide structure. The shoulder at 1160 cm^−1^, especially for sample RHA2, is related to the formation of Si–O–C bond^31^. The band intensities at 800 and 461 cm^−1^ reflecting the Si–O–Si stretching and wagging vibration of silica, respectively^32^. These findings suggest that RHA1, RHA2, and RHA3 could be useful as precursors for the fabrication of carbon/silica nanocomposites.

### Activation mechanism

KOH breaks the C-H bond, causing the carbon compounds in RHA to form and exfoliate, and then transition into graphene oxide, which has a large surface area and great removal properties^16^. Chemical activation by KOH at 850 °C results in a rise in carbon and oxygen content, as well as partial breakdown of volatile chemicals and degradation of organic components, yielding high pure carbon (as described below). Although the KOH activation mechanism is a complex process involving many chemical reactions, it may be broken down into three steps based on the temperature range of the chemical activation processes. For RHA carbon content, the first step occurred at temperatures lower than 700 °C in the presence of KOH, as shown in the Eq. (1)^33^.

1 $$6{\text{KOH}} + 2{\text{C}} \to 2{\text{K}} + 3{\text{H}}_{2} + 2{\text{K}}_{2} {\text{CO}}_{3}$$

At temperatures above 700 °C, the second stage of the activation process by KOH begins to dissolve into K_2_O and CO_2_ (Eq. 2) and eventually decomposes completely at temperatures above 800 °C. Furthermore, carbon has reduced K_2_CO_3_ and K_2_O to form free metallic potassium (K) (Eq. 3) and (Eq. 4)^34^. Furthermore, amorphous silica removed from rice husk ash due to reaction between K_2_O and SiO_2_ in this step of activation mechanism (Eq. 5)^33^.

2 $${\text{K}}_{2} {\text{CO}}_{3} \to {\text{K}}_{2} {\text{O}} + {\text{CO}}_{2}$$

3 $${\text{K}}_{2} {\text{CO}}_{3} + 2{\text{C}} \to 2{\text{K}} + 3{\text{CO}}$$

4 $${\text{K}}_{2} {\text{O}} + {\text{C}} \to 2{\text{K}} + {\text{CO}}$$

5 $${\text{K}}_{2} {\text{O}} + {\text{SiO}}_{2} \to {\text{K}}_{2} {\text{SiO}}_{3}$$

When the temperature rises above 800 °C, metallic potassium melts and penetrates the graphitic layers of RHA, resulting in layer exfoliation and an increase in surface area. When potassium is removed by washing with distilled water, the effect on carbonaceous layers is irreversible^35^.

In addition to the temperature of activation, the weight ratio of potassium hydroxide to RHA might be used to adjust the degree of RHA activation. The amount of metallic potassium exfoliated from the carbonaceous matter increases as the ratio of KOH to rice husk ash increases, and the specific surface area increases^36^. The 5:1 ratio was used in this investigation based on Muramatsu and co-workers' experimental results from the synthesis of stable GO sheets from RHA with excellent purity^37^. The effective removal of amorphous carbon and the strong penetrating behavior of molten KOH into crystalline graphene are two crucial roles of KOH in the production of graphene sheets from RHA.

### Characteristics of FGS nanocomposites after activation of RHA

KOH activation has been shown to be an effective method for converting RHA as carbonaceous precursors to FGS, considerably improving the chemical and physical properties and, as a result, the adsorption qualities.

#### Phase features

**Figure 3:**
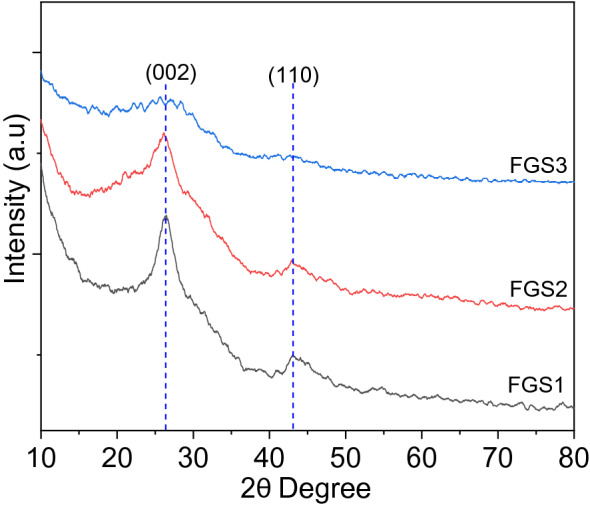
XRD pattern of functionalized graphene oxide/SiO_2_ nanocomposites.

XRD was used to analyze the crystal structure of FGS samples, as shown in Fig. 3. The large peak at 26.2 and 43.3° attributable to (002) and (110) GO pattern reflections in sample FSG1 and FSG2 suggests graphite expansion and exfoliation in a few GO layers and might be indexed to the GO [JCPDS 25-284], and it is compatible with graphene data^37^. Generally, the peaks of SiO_2_ is not observed because it mainly amorphous^32^. The XRD agree with the previous studies and also compatible with HR-TEM observations (as described below). The crystallinity of samples obey the order FGS1 > FGS2 > FGS3, taking into account the silica is amorphous. As a result, the absence of broad peaks in sample FSG3 is attributed to the composite's increased silica concentration.

The number of layers was estimated according to the method in supplementary information S-4. The number of layers is approximately 11 and 10 layers for sample FGS1 and FGS2, respectively (Table 1). On the contrary, we cannot estimate the number of layers in sample FGS3 because the band at 002 is not observed^38^.

**Table 1:** Physicochemical characterization of GO/SiO_2_ nanocomposites.

Sample	d (Å)	Number of layers	S_BET_ (± 5 m^2^ g^−1^)	S_micro_ (± 5 m^2^ g^−1^)	L_0_ (± 0.1 nm)
FGS1	3.376	11	1390	2398	2.64
FGS2	3.411	10	1768	2499	3.55
FGS3	–	–	1255	1631	2.39

#### Surface morphology

**Figure 4:**
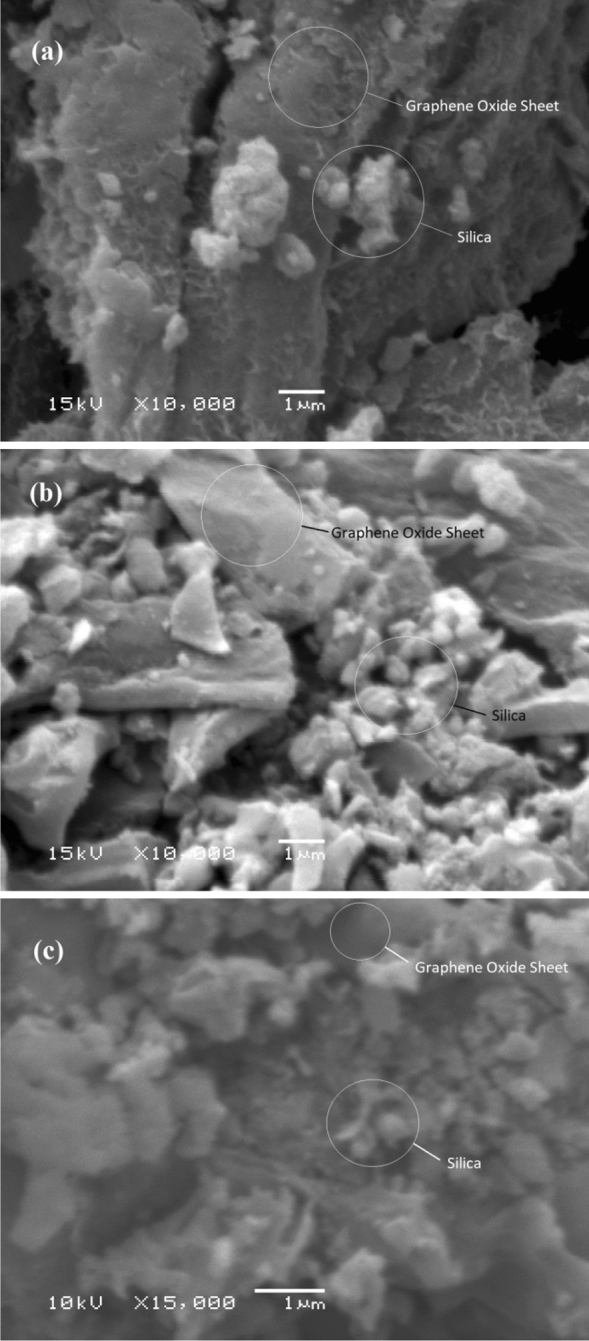
SEM images of prepared graphene oxide/SiO_2_ nanocomposites: (**a**) FGS1, (**b**) FGS2 and (**c**) FGS3.

Examining the shape of silica nanoparticles on the surface of a GO nanosheet is the most basic way to determine their attachment impact. SEM was used to examine the surface morphology of graphene oxide/SiO_2_ nanocomposites created by RHA activation treatment with KOH. Figure 4 shows SEM images of the examined composites FGS1, FGS2, and FGS3. The presence of oxygen-containing functional groups is indicated by the creation of many wrinkles in the structure of GO^23^. These wrinkles are considered to be high-energy adsorption sites. There are changes in the impregnation of SiO_2_ on the surface of GO nanosheets between three samples. The number of silica nanoparticles appears to rise in the following order: FGS1 > FGS2 > FGS3. At sample FGS3, dense silica particles are clearly deposited on the GO sheets (Fig. 4c).

**Figure 5:**
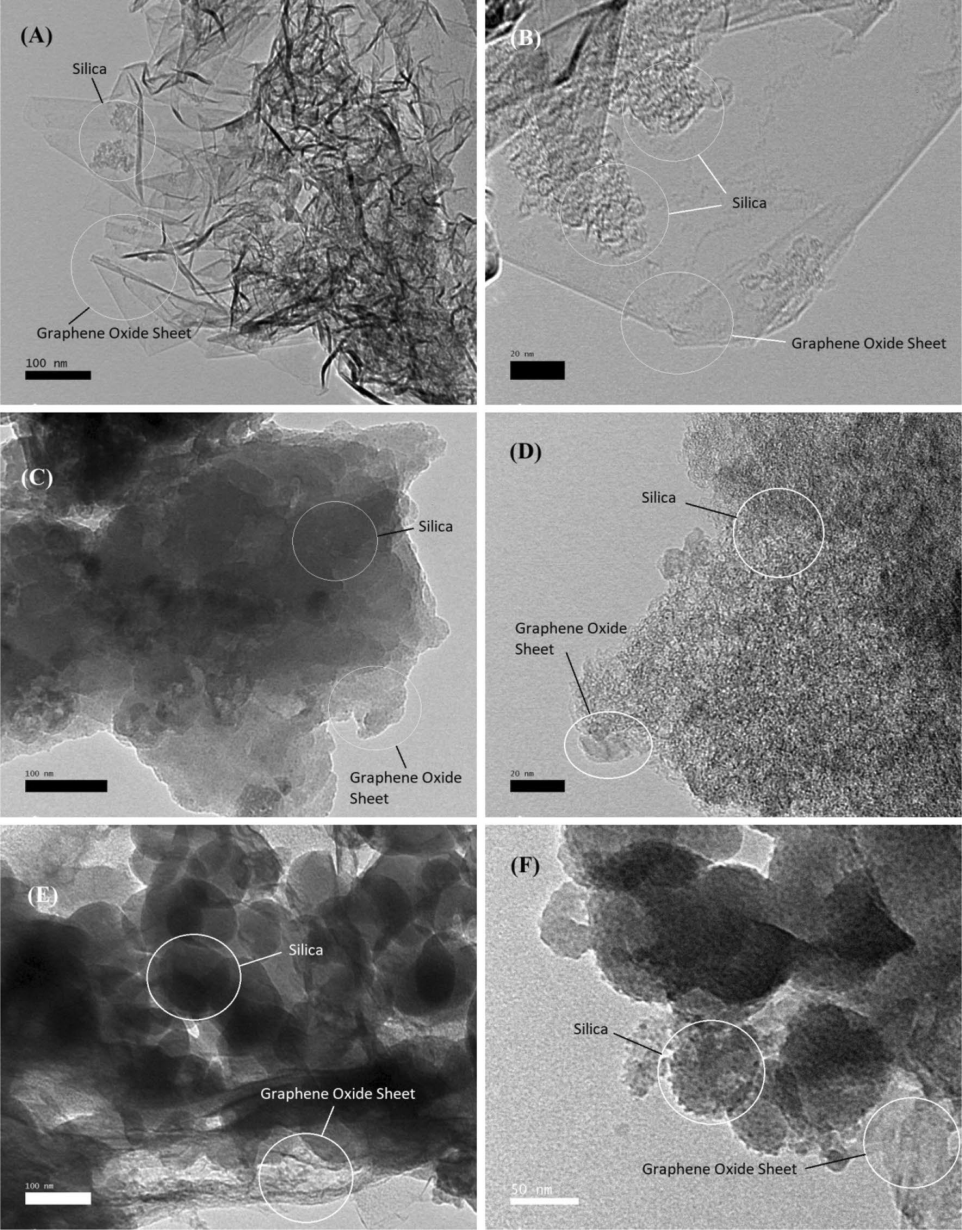
TEM images of prepared GO/SiO_2_ nanocomposites: (**A**,**B**) FGS1, (**C**,**D**) FGS2, and (**E**,**F**) FGS3.

Further observation for the morphology of graphene oxide/silica samples have been explored deeply by HR-TEM microscopy and the images are presented in Fig. 5. As we know, the graphene was highly aggregated and presented a blocky structure^15^. In general, the sheet architecture is related to the graphene oxide structure, while the spherical structure attributed to SiO_2_. TEM analysis was provided, the changes are made on the surface of graphene oxide sheets are dramatic and clearly visible. In high magnification, Fig. 5a indicates that the sample FSG1 exhibits the highest degree of exfoliation. It was consisted of thin and transparent layers with some wrinkles on the surface of GO nanosheet indicating the oxygen containing groups on the GO. Moreover, the low amount of silica nanoparticles was obvious in the low magnification (Fig. 5b), demonstrating the successful preparation of functionalized GO/SiO_2_ nanocomposites. In FGS2, the silica nanoparticles were covered the GO nanosheet which indicate the increase the percentage of silica that it is obvious in Fig. 5c,d. In higher magnification, a lot of nanospheres are observed in the image of FGS3 (Fig. 5e,f), confirming the existence of silica nanoparticles on the surfaces of GO sheets.

The inclusion of silica nanoparticles works as a spacer to prevent FGS samples from re-stacking, particularly for FGS3. At different magnifications, spherical and uniform silica nanoparticles on the surface of the GO nanosheet can be seen (Fig. 5e,f). Rise in loaded silica nanoparticles on the surface of GO with order FGS1 > FGS2 > FGS3, showing that increasing the temperature of manufacturing RHA leads to an increase in the percentage of silica and a reduction in the degree of exfoliation, as shown in Fig. 5. The interaction of silica nanoparticles with two-dimensional (2D) GO nanosheets could result in the creation of a silica GO nanohybrid and the separation of the stacked interlamination of graphene, thus inhibiting nanoparticle aggregation^39^. The SiO_2_ nanoparticles, not the graphene oxide nanosheets, clearly influenced the surface morphologies of the FGS samples. To summarize, the major morphology in FGS3 is SiO_2_ nanosphere, whereas the main morphology in FGS1 is GO, and in FGS_2_ the main morphology is a sheet structure covered in silica nanoparticles.

#### Fundamental composition

**Figure 6:**
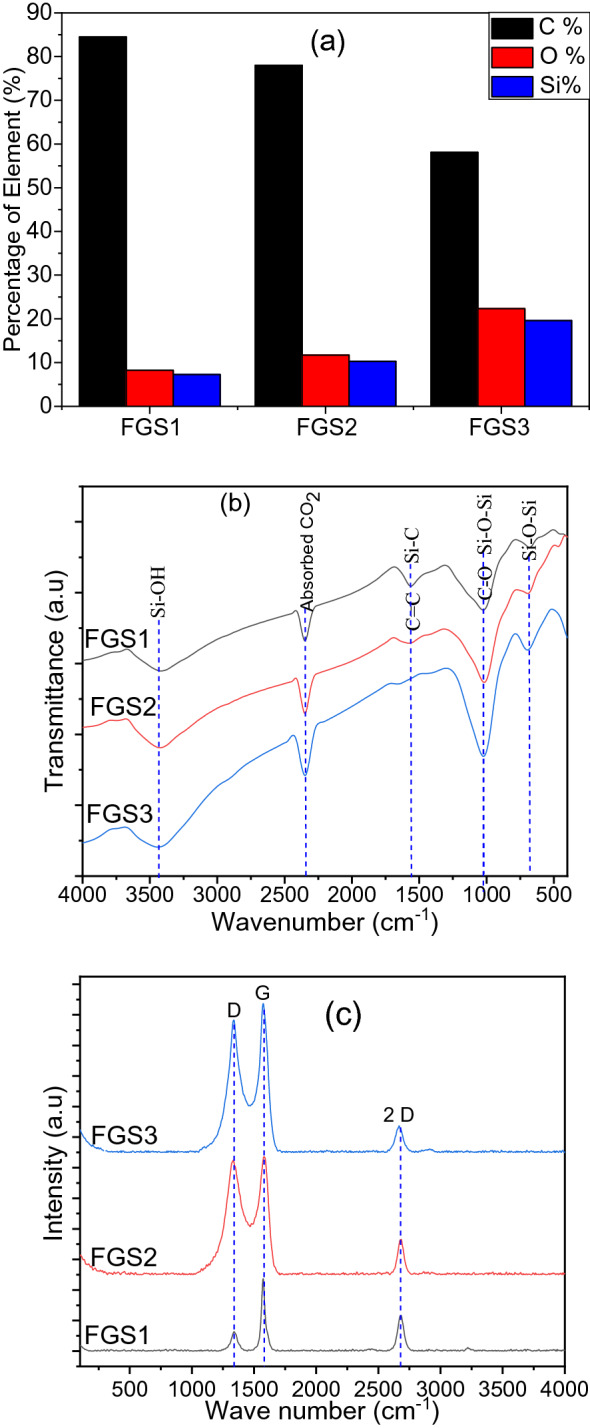
(**a**) Chemical composition, (**b**) FTIR, and (**c**) Raman of GO/SiO_2_ nanocomposite samples.

Using a combination of EDX, FTIR, and Raman, the graphitic nature of the materials was demonstrated. The carbon content of FGS is larger than that of generated RHA, indicating that activation has a positive effect. KOH destroys the C-H bond, allowing amorphous carbon to develop quickly^37^. From Figs. 2a and 6a, As a result of KOH's effect on boosting the percentage of C %, the results show a significant increase in C % and a decrease in Si percent. i.e. C % increase from 49.09% for sample RHA1 to 84.48% for sample FGS1. The highest oxygen level measured in combination with the highest silicon content in sample FGS3 indicates an increase in silica. Because of the production of silica, the C content of FGS3 is lower than that of FGS1. Also, it reveals that the C/O ratio in the FGS1 (10.22) is higher than that in the FGS2 (6.65) and FGS3 (2.60). The amorphous silica content of RHA was not totally eliminated after activation by KOH, and it increased as the ratio of silica in the precursor increased, according to XRD and HR-TEM studies. As shown in Fig. 2a, the silica content of rice husk increased as the burning temperature increased, and more amorphous silica remained after activation. These findings were in line with the literature^40^.

The oxidation state and functional groups of FGS samples were validated by FTIR and reported in Fig. 6b in agreement with the elemental analyses. The large peak at 3446 cm^−1^ represents the hydroxyl functional group's O–H stretching vibration as well as Si–OH bending. Furthermore, the FGS nanocomposites are distinguished by the emergence of C–Si stretching in the region of 2349 cm^−1^, which confirms the GO surface in nanocomposites^16^. The peak at 1554 cm^−1^ is attributed to the band of SiC lattice and also related to the bound H_2_O molecules^15,41^. In addition, the band within the 1013–1026 cm^−1^ range is caused by identical oxygen atom vibrations in the Si–O–Si linking direction and also may be related to the alkoxy bands originating from the functional groups on the surface of GO^15,23^. Finally, the peak at 683 cm^−1^ corresponds to silica group symmetric stretching deformation. The intensity of the silica and silica–carbon bands increases in the following order: FGS1 > FGS2 > FGS3, showing improved silica integration onto the GO surface layers, given that silica is primarily present in sample FGS3.

The FTIR spectra of all samples show all of the bands ascribed to oxygen-functional groups, showing the presence of functional oxygenated groups in the graphitic structure, silica network, or both. As a result, silanol groups appear to allow anchoring on the sp^2^-sp^3^ deficiencies^31,40,42^, promoting the successful assembly of SiO_2_ and GO into FGS samples. The carboxyl and/or hydroxyl groups of GO and the hydroxyl groups in the silanol groups on the FGS surface formed a condensation process. As a result of the heat activation process, covalent ester bonds were generated^43^. The addition of silane groups to FGS samples improves hydrophilicity and lowers resistance between the liquid solution and the solid adsorbent surface. Furthermore, the hydroxyl, carboxyl, and epoxy functional groups of FGS have a negative charge, which aids in the formation of bonds with anionic dyes like Trypan blue.

Figure 6c displays the Raman spectra of FGS1, FGS2, and FGS3 samples. These results are in line with previous studies on the Raman spectra of graphene^44,45^. As we know, pure silica has not any obvious peak in the Raman spectra^37^. All spectra shows the main characteristics at Raman D (1347 cm^−1^) and G (1574 cm^−1^) bands of the graphitic samples, which arisen from the structure defects or disorder vacancies and the in-plane vibration of aromatic sp^2^ carbon (C=C) of GO, respectively^44^. When compared to previous studies, the slight shift in the wave number of the bands for samples FGS is due to charge transfer between GO and SiO_2_, which is an evidence of the electrostatic interaction between the two components^39^. The G band revealed a graphitic structure in the synthesized sample that was responsive to strain effects and GO layers. In addition, the amount of graphene layers present in the sample has a direct relationship with the position of frequencies shift in Raman spectra. Furthermore, the presence of a prominent 2D-band at 2667 cm^−1^ indicates that highly crystalline 2-dimensional GO is growing and has a lamellar structure^42^. For all products, the spectra strength of G band is higher than of D band^13^. The I_D_/I_G_ ratios from Raman spectra for FGS1, FGS2, and FGS3 were determined to be 0.26, 0.98, and 0.89, respectively, in this work. The highest graphitization degree coupled with the highest degree of oxidation during the synthesis and limited structural order within the graphitic carbon network is revealed by sample FGS2 (I_D_/I_G_ = 0.98)^44^. FGS1, FGS2, and FGS3 had I_2D_/I_G_ intensity ratios of 0.42, 0.26, and 0.18, respectively, which clearly indicates the formation of double or few layers of exfoliated GO.

Raman analysis, thus, agrees with the FTIR, as confirming the graphitic nature of the sample FGS2 and furthermore declares the highest oxidation degree of this sample.

#### Textural characteristics

**Figure 7:**
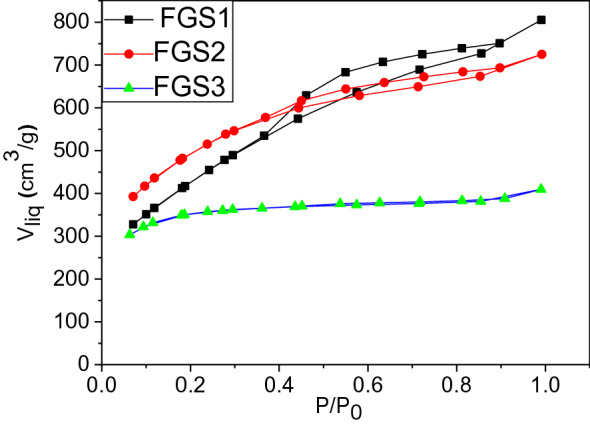
N_2_ adsorption isotherms of functionalized GO/SiO_2_ nanocomposites.

Figure 7 shows the textural features of graphitic composites as determined by N_2_ adsorption isotherms. The shape of the isotherms already indicates that all samples have different porosities; however, micropore and mesoporous structures can be seen. Both FGS1 and FGS2 showed type-IV adsorption isotherms characteristics of micoporous-mesoporous materials, while mainly type-II isotherm was obtained for sample FGS3.

In the low-pressure region, the sample exhibited a steep increase in nitrogen uptake due to the presence of micropores. When P/P_o_ exceeded 0.4, the isotherm revealed a hysteresis loop, which indicated the capillary condensation of mesoporous structure. When comparing the isotherms of samples (Fig. 7 and Table 1), a different porous texture due to the interactions and transformations between GO and SiO_2_ is clearly visible. Thus, the surface area of FGS1 form RHA at 400 °C (S_BET_ = 1390 m^2^ g^−1^) is quite high, while FGS3, at 600 °C, presented a low surface area (S_BET_ = 1255 m^2^ g^−1^), associate to the developed of mesoporosity during the activation process by the increasing the percentage of SiO_2_ and reduced the percentage of carbon. In fact, the sample FGS2 is the highest surface area (S_BET_ = 1768 m^2^ g^−1^) because it is mainly microporous structure as well as the perfect exfoliation of GO, while the lowest surface area in FGS3 related to the increase the percentage of silica nanoparticles and decrease the percentage of GO (observed at HR-TEM and XRD).

The interaction of carbon with silica is confirmed by the change in surface area. The microporosity of all composites is preserved in the shape of the isotherm. The regulated crystal development of SiO_2_ crystals in tandem with the established structure of microporous carbon is responsible for FGS2's maximum surface area. This demonstrates that the GO/SiO_2_ composites are porous, with the pores in FGS3 being predominantly mesoporous. The surface area results are all higher than the previously reported values^39,42^.

### Growth mechanism

**Figure 8:**
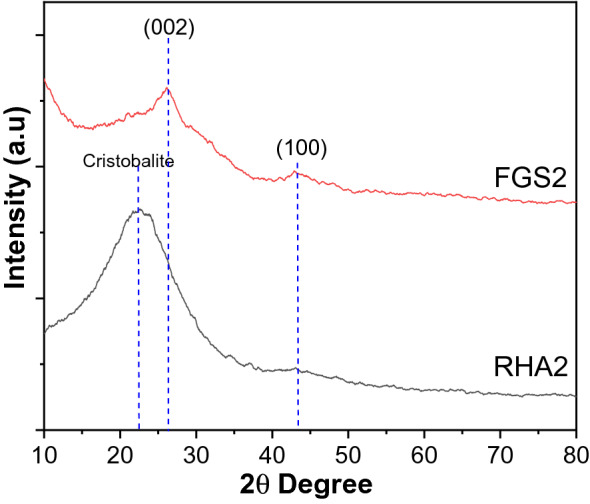
XRD of RHA2 and FGS2.

In RHA2, rice husk ash at temperature 500 °C, the diffraction patterns the diffraction patterns of graphene is weak or even disappear and only broad peak at 15–30° (22–23°) matching well with Cristobalite [JCPDS 29-0085]^46^. It indicates the existence of silica without any conversion of graphitic structure into graphene materials (Fig. 8). The development of RHA2 to crystalline graphene was confirmed by X-ray diffraction patterns after activation with KOH at a higher temperature, indicating that the silica impurities in RHA were effectively removed as a result of the elimination of the broad silica peak (Fig. 8). Exfoliation disrupted the regularly stacked graphitic structure, as evidenced by the weak (002) and (100) peaks detected in graphene oxide, which is further supported by HR-TEM findings (Fig. 5). The formation of oxygen-containing functional groups in the basic plane of natural graphite was attributed to the crystalline structure of graphite being disrupted in RHA2, resulting in this observation^47^.

Epoxy, carboxylic acid, and hydroxyl groups are some of the reactive oxygen functional groups found in GO. It was successfully functionalized and reduced by mesoporous silica in this study by activating rice husk ash with KOH at autoclaving temperature and then heating to 850 °C. Exploiting the functional groups of GO such as hydroxyl and carboxylic acid and the silanol (Si–OH) and siloxane (Si–O–Si) groups in silica for the formation of the Si–O–C bond has not been reported yet. The possible suggested routes for the reaction are shown below:

6 $${\text{G}}{-}{\text{OH}} + {\text{Si}}{-}{\text{OH}} \to {\text{G}}{-}{\text{O}}{-}{\text{Si}} + {\text{H}}_{2} {\text{O}}$$

7 $${\text{G}}{-}{\text{COOH}} + {\text{Si}}{-}{\text{OH}} \to {\text{G}}{-}{\text{COO}}{-}{\text{Si}} + {\text{H}}_{2} {\text{O}}$$

Silica was functionalized onto the GO surface during the activation step, which requires COOH as a conjugating moiety. The epoxy and hydroxyl groups make up the majority of the oxygen functionalities of GO, while carboxyl groups make up just a minor portion of the oxygen functionalities^48^. To optimize the proportion of silica on the GO sheet, the hydroxyl groups were changed to carboxyl acid moieties before activation^49^. So, according to the result of XRD, FTIR, and HR-TEM, it indicates that silica particles grew gradually on the surfaces of GO nanosheets when the regular stacks of GO were completely destroyed. Meanwhile, the newly obtuse peak at 22-23° demonstrates that the silica nanoparticles are amorphous.

**Figure 9:**
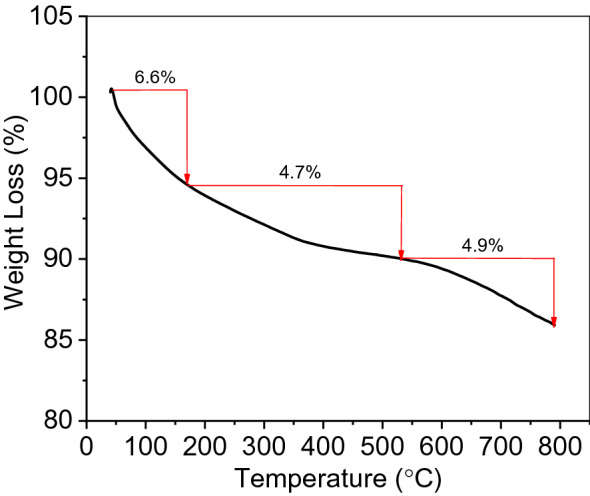
Thermal stability behavior of FGS2 by TGA analysis.

Another evidence for the growing the silica on the surface of GO is obtained by TGA. The thermal stability of GO/SiO_2_ nanocomposites, as well as the presence of silica, was demonstrated by thermogravimetric analysis (TGA). TGA was conducted under nitrogen and at room temperature to 800 °C with a heating rate of 5 °C min^−1^. In Fig. 9, three stages could be identified in the mass loss curve of GO/SiO_2_ nanocomposites. In details, slight mass loss (~ 6.6%) upon thermal decomposition to 164 °C which represented the evaporation of water adsorbed on the surface in the GO/SiO_2_ sample. The second stage happened between 164 and 540 °C and beyond this temperature the sample loses only 4.7% in this stage which was the decomposition of oxygen-containing functional groups (phenol, epoxy, and alkoxy) in GO nanosheets. In the third stage (540–800 °C), mass loss was due to the sublimation and gasification of carbon structure in the GO composite. The thermal stability of GO/SiO_2_ is very high as indicated the existence of silica and also decreased the oxygen-containing functional groups as a result of combination it with silica. As a result, the impregnation of silica is responsible for the increasing the thermal stability of GO which is an agreement with the literature^23,50^. Furthermore, the residue of GO/SiO_2_ at 800 °C was 85.9% which is much higher than that for GO in previous studies (normally less than 50%) under the same conditions, indicating the presence of silica on GO^50^.

Related to the silica content, it was determined by heating the optimized sample, FGS2 in air and beyond this temperature and the sample loses only 88.57% of its weight as the temperature increased to ~ 1200 °C, presumably due to pyrolysis of all graphitic structure and the percentage of silica nanoparticles was 11.42%. These data was supported with the results of HRTEM in Fig. 5 and FTIR in Fig. 6b.

### Adsorption studies towards TB or BPA

#### Reactivity of FGS samples

**Figure 10:**
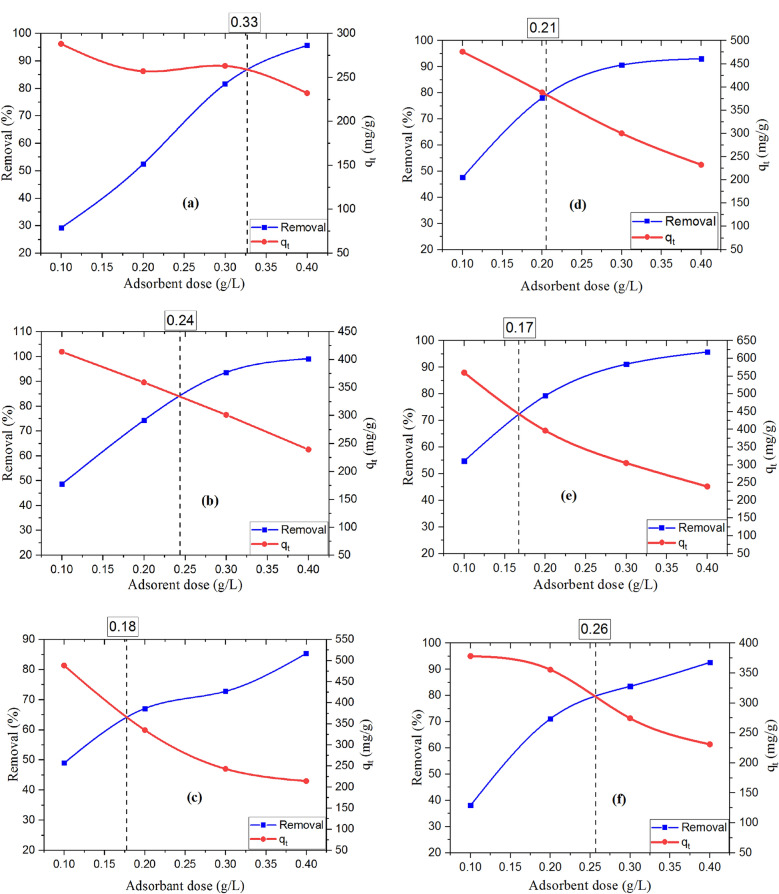
Effect of dose on the adsorption of TB onto (**a**) FGS1, (**b**) FGS2, and (**c**) FGS3, and on the adsorption of BPA onto (**d**) FGS1, (**e**) FGS2, and (**f**) FGS3 [pH: 3, initial concentration:100 ppm, agitation speed: 300 rpm, contact time 30 min, and temperature 25 °C].

From Fig. 10 and Table 2, the results explained that the order of TB adsorption per unit mass (*q*) was FGS2 > FGS3 > FGS1, where for BPA it was FGS2 > FGS1 > FGS3. As a result, FGS2 was the best composite for removing both TB and BPA on a mass (mg/g) and percent of removal scale, while all composites removed BPA more efficiently than TB. On the other hand, the lowest efficiency in this study is FGS1 for TB removal, as the results show that the smallest q value with the largest adsorbent dose value belongs to FGS1, implying that larger quantities of adsorbent are required and the reaction is not cost-effective when compared to other products.

**Table 2:** The optimum adsorbent dose and q_t_ after 30 min for FGS1, FGS2, and FGS3.

Adsorbent	TB			BPA		
	FGS1	FGS2	FGS3	FGS1	FGS2	FGS3
Optimum dose (g/L)	0.33	0.24	0.18	0.21	0.17	0.2
*q*_*t*_ (mg/g)	255	376	276	368	414	321

#### Effect of adsorbent dose

Figure 10 depicts the association between the adsorbent dosage of FGS samples and the adsorption of TB or BPA. Table 2 also shows the influence of FGS sample dose on the highest amount and removal efficiency of TB and BPA after 30 min with the optimal adsorbent dose of graphene nanocomposites (FGS1, FGS2, and FGS3). The removal efficiency improved overall when the adsorbent dose was increased from 0.1 to 0.4 g/L. This is due to the increased surface area of the adsorbent and the availability of extra adsorption sites. As demonstrated in Fig. 10, the amount of TB or BPA absorbed by FGS nanocomposites by unit weight (q_t_) decreased as the adsorbent mass increased. Because adsorption sites remained unsaturated during the adsorption reaction, the amount of pollutant absorbed decreased as adsorbent mass increased. However, with increased exposure over a certain dose, the increase in pollutants elimination is insignificant. This is because a larger adsorbent dosage causes faster surface adsorption on the adsorption sites, resulting in a lower concentration of the adsorbate in the solution than a lower adsorbent dose. As a result, as the dose of adsorbent is raised, the quantity of pollutants adsorbed per unit mass of adsorbent decreases, resulting in a fall in the value of q_t_^51,52^.

#### Effect of contact time and adsorption kinetics

On the optimized adsorbent FGS2, the effect of contact duration on the adsorption of TB and BPA was investigated at varied initial dye concentrations, as shown in Fig. S2a,b, respectively. The vast majority of the TB or BPA was clearly adsorbed after 30 min, followed by a more progressive sorption process until adsorption equilibrium was reached. The rapid sorption rate observed in the first stage can be attributed to the abundance of available adsorption sites on the FGS2 surface due to Vander Walls and electrostatic forces, as well as rapid diffusion to the adsorbent surfaces, whereas the slow sorption rate observed in the second stage can be attributed to the fact that the vast majority of available active sites on the sorbent surface are occupied by adsorbates, and the sorption process that needs to occur further down the line^53^.

Adsorption kinetics, as is well known, may provide a wealth of information for confirming the composites' prospective application value and aiding in a thorough understanding of the adsorption process. Because the exact mechanism was unclear, then a series of models were introduced. Our data were fitted by the pseudo first-order (PFO) and pseudo-second-order models (PSO). The fitting data were tabulated in Table S3, and fitted lines towards TB and BPA elimination on FGS2 were showed in Fig. S2.

The validity of the pseudo-first order (PFO), pseudo-second order (PSO), intraparticle diffusion, and Boyd models for TB and BPA adsorption onto FGS2 adsorbent was assessed in this study. These kinetics models aid in the design of solid/liquid sorption processes and mechanisms by examining rate-controlling phases such as mass transport and chemical reaction processes using four kinetic models. The kinetics of both TB and BPA onto the produced FGS2 were described using the linear forms of both models (Table S2).

Despite the strong R^2^ values, the mismatch between q_e_ and q_exp_ values in Fig. S2 and Table S2 suggested that the PFO model does not describe the adsorption of TB and BPA onto FGS2 from aqueous solutions. On the other hand, the experimental adsorption capacity (q_exp_) was in agreement with the calculated adsorption capacity (q_e_ calculated) from the PSO model, and the R^2^ of the PSO model exceeded 0.99, which was significantly higher than that of the PFO model, indicating that FGS2 chemisorption towards TB and BPA, indicating that the chemical adsorption is the rate-controlling phase in the adsorption process, and the adsorption mechanism is influenced by a range of factors (e.g., electron donor–acceptor interaction, H-bond, and so on).

#### External mass transfer and diffusion model analysis

Increased agitation speed promotes turbulence in the solution, lowering the thickness of the external boundary film in batch adsorption systems. As can be seen from Fig. S3a,b, TB and BPA removal efficiency increased from 9.1 to 48.57 and from 24.5 to 54.8% with the agitation speed increased from 0 to 300 rpm at adsorbent dose 0.1 g/L at initial concentration of 100 mg/L, respectively. Therefore, the external diffusion resistance is constant and can be neglected at agitation speed higher than 300 rpm.

Adsorption and equilibrium state attainment are primarily caused by bulk diffusion, (1) in which adsorbate diffuses from bulk solution to the boundary layer of the liquid film adjacent to the adsorbent, (2) film/surface diffusion, in which adsorbate diffuses through the liquid film, and (3) intraparticle (pore) diffusion, in which adsorbent carry the available pore sites because pores increase surface area and thus improve film/surface diffusion (as described below). Because a sufficient speed of shaking was used during the adsorption, bulk diffusion can be ignored. Weber and Morris devised a kinetic model to represent the time-dependent intraparticle diffusion of nanoparticles. Weber and Morris developed a kinetic based model that symbolizes the time dependent intraparticle diffusion of adsorbate^20^.

**Figure 11:**
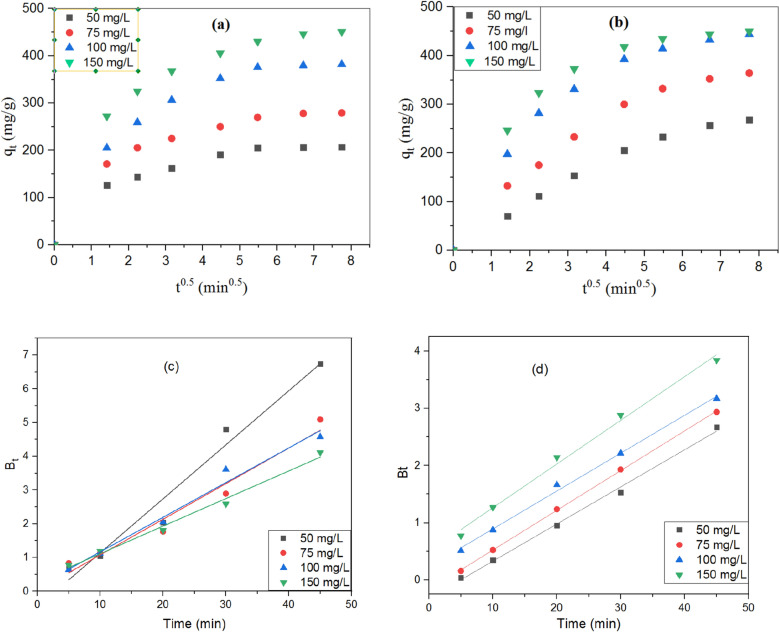
Intraparticle diffusion plots for removal of (**a**) TB, and (**b**) BPA, and Boyd plots for removal of (**c**) TB, and (**d**) BPA at initial concentrations using FGS2 sample [pH: 3, agitation speed: 300 rpm, and solution temperature: 25 °C].

To further comprehend the adsorption mechanism, the intraparticle diffusion model was used to figure out the process of diffusion. From Fig. 11a,b, it revealed that there are two stages the adsorption process. The first stage, sharper region, fastest step, revealed that TB or BPA gradually occupied the active location of FGS2's surface that transported from bulk solution (Film diffusion). The second stage, the TB or BPA entered into the interior pores by intra-particle diffusion, as reflected by the second linear part of the plot (Intraparticle diffusion). As a result, intraparticle diffusion has become the most important element in determining the rate of adsorption at this stage. From Fig. 11 and Table 3, the values of C ≠ 0 and different values of k_in_ indicate the intraparticle diffusion is not the only the rate controlling step and additional reactions by chemical forces such H-bond, may be attributed to the principal mechanism of action in addition to intraparticle diffusion.

**Table 3:** Parameters of intraparticle diffusion model for removal of TB and BPA using FGS2 at different initial dye concentrations [pH: 3, agitation speed: 300 rpm, and solution temperature: 25 °C].

	Initial TB concentration (mg/L)					
	*k*_*d1*_	*C*_*1*_	R^2^	*k*_*d2*_	*C*_*2*_	R^2^
**TB**						
50	51.32	20.59	0.8773	4.42	175.51	0.6315
75	75.03	27.78	0.8837	8.56	217.00	0.8313
100	97.51	26.73	0.9380	8.35	321.52	0.7544
150	116.56	42.09	0.8980	13.66	349.89	0.9159
**BPA**						
50	48.71	0.76	0.9996	19.22	123.68	0.9693
75	73.19	10.52	0.9818	19.22	219.98	0.9510
100	106.79	20.86	0.9633	15.49	326.42	0.9798
150	119.57	31.99	0.9388	9.54	378.23	0.9423

According to Table 3, the increased driving force that facilitates intra-particle diffusion of the TB or BPA onto FGS2 causes kd1 to increase with increasing the initial TB or BPA concentration. Furthermore, the thickness of the boundary layer in the second region, which is related to intraparticle diffusion (C2), was larger than in the first region, which is related to film diffusion (C1).

It was further investigated by Boyd model that determined the actual slow step in the adsorption process for solute transport in order to discover the real rate-controlling step for adsorption of both TB and BPA onto the produced FGS2 and to solve the issue of dual nature of intraparticle diffusion (film and pore diffusion). Figure 11c,d indicated the plot of B_t_ versus t for adsorption of TB and BPA from polluted water, respectively. As a result, the straight lines did not pass through the origin, showing that intraparticle diffusion was not just a rate-controlling step, but also a combination of external diffusion, intra-particle diffusion, and chemical reaction might be the primary transfer mechanism^49^.

#### Effect of initial dye concentration and equilibrium isotherms

The concentration of pollutant can be eliminated decreases as the concentration of the pollutant rises. Despite this, the real quantities of TB or BPA adsorbed per unit weight (mg/g) on FGS2 rose with higher initial concentrations and enriched the equilibrium after 30 min (Fig. S2), which could be due to the significant mass transfer driving forces at high initial TB and BPA concentrations^12^. When the initial concentration of TB dye increased from 50 to 150 mg/L, the actual amount of TB and BPA adsorbed per unit mass increased from 205.82 to 430.15 and the percentage of removal decreased from 98.08 to 73.52%. On the other hand, the removal results (q) of BPA was increased by unit mass from 233.03 to 434.39 and on scale of removal % decreased from 77.10 to 48.51%, respectively.

Adsorption isotherms were used to determine the distribution of adsorbates between the liquid and solid phases. If the amount of solute adsorbed by the adsorbent is equal to the amount desorbed, the adsorption process is said to be in equilibrium^52^. Equilibrium isotherms are constant values that indicate the surface characteristics and sensibility of the adsorption equilibrium of the adsorbent that forms when the adsorbate's liquid phase concentration is in equilibrium with the adsorbent's solid phase. Adsorption isotherms depict qualitative information regarding solution surface interaction mechanics and the critical relationship between adsorbate concentration and adsorption site accumulation rate at a constant temperature.

To study the equilibrium performance of GO/SiO_2_ hybrid nanocomposites for eliminating TB and BPA from aqueous solutions, the findings of the experimental equilibrium were analyzed using Langmuir, Freundlich, and Temkin isotherms (Fig. S4). It showed that for both TB and BPA, the sample FGS2 obeyed adsorption according to the Langmuir model. From Fig. S4 and Table S3, the Langmuir model is the best adsorption isotherm model for removal of TB and BPA by FGS2, and the correlation coefficient is ≥ 0.99. Furthermore, all removal trials of TB and BPA had values of q_m_ that were extremely similar to the experimental removal capacity (q_e_ from Table S3), supporting the Langmuir adsorption isotherm model in interpreting the data. The estimation of separation factor (R_L_) is presented in supplementary information S-5S-5. Table S4 shows the R_L_ values for the adsorption of TB and BPA on FGS2 nanocomposites. The Langmuir isotherm was shown to be appropriate for TB and BPA adsorption at all concentrations studied, with all R_L_ values falling between zero and one.

In comparison, the correlation coefficient (R^2^) values of the Freundlich isotherm demonstrated that the Langmuir isotherm must be the most suited isotherm for explaining the experimental data at equilibrium for FGS2 nanocomposites TB and BPA adsorption (Table S3). In the Temkin isotherm model, adsorption is defined by a uniform distribution of binding energies up to a maximum binding energy^54^. According to the Temkin isotherm derivation, the decrease in adsorption heat is linear rather than logarithmic, as the Freundlich equation suggests. Table S3 shows that the KT values for both BPA and FGS2 were extremely low. The Temkin isotherm was also shown to be inadequate for BPA adsorption, while it was found to be suitable for TB adsorption by FGS2.

In summary, the Langmuir, Freundlich and Temkin isotherm models adequately describe the adsorption equilibrium of TB and BPA on FGS2 (Table S3). The validity for the given data of the three isotherm models roughly conform the order of Langmuir isotherm > Freundlich isotherm >  > Temkin isotherm, for all cases of removal of TB and BPA on FGS2.

#### Effect of Temperature and adsorption thermodynamics

**Figure 12:**
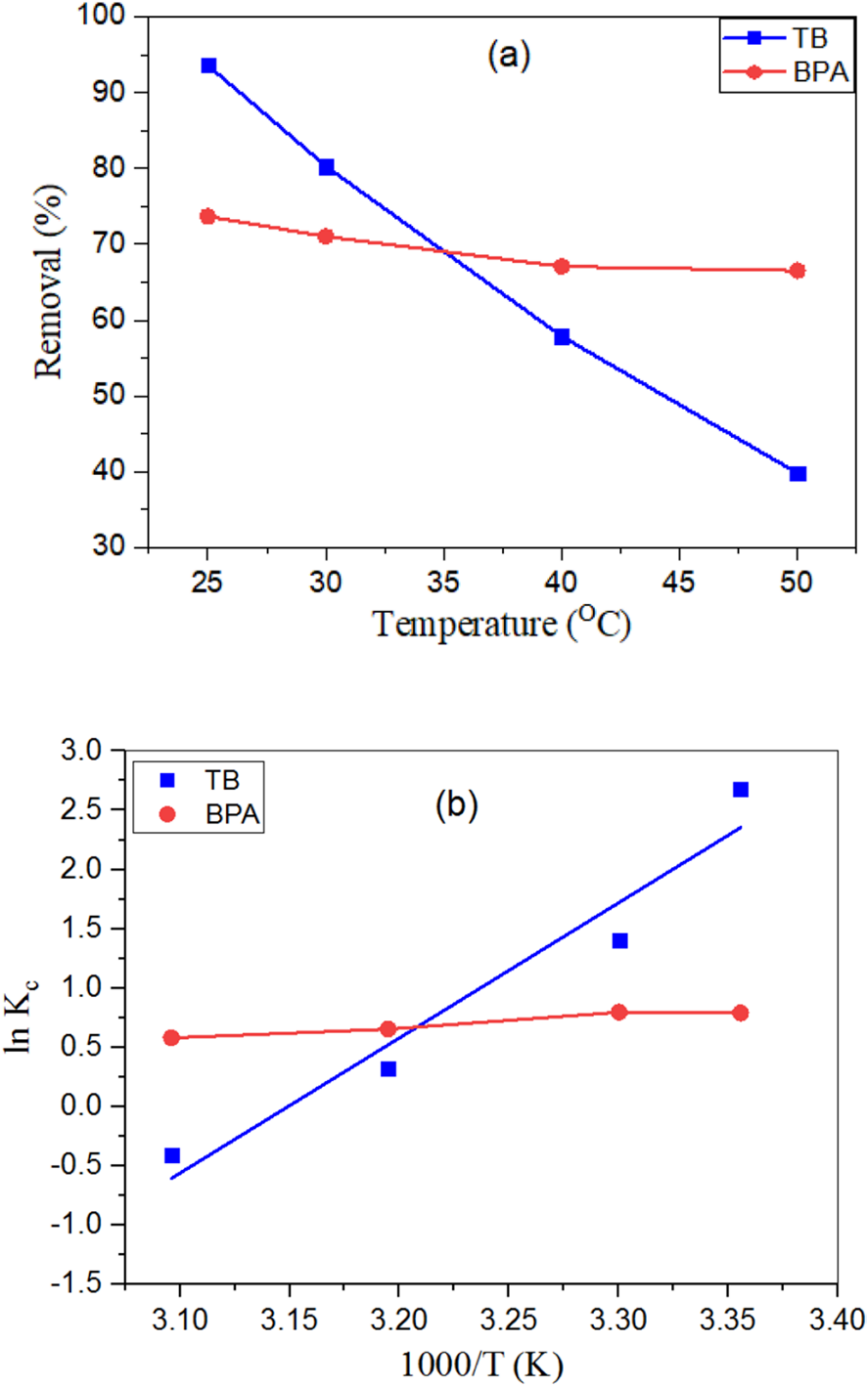
(**a**) Effect of temperature on efficiency of adsorption of TB and BPA onto FGS2, (**b**) Plot of lnk2 vs. 1/T, (**c**) Van't Hoff plot for TB and BPA removal using FGS2 composites at different solution temperatures [pH: 3, agitation speed: 300 rpm, contact time 30 min and optimum adsorbent dose].

Experiments were carried out at various temperatures, including 25°, 30°, 40°, and 50 °C, to investigate the temperature effect of the FGS2 on the elimination of TB and BPA, as shown in Fig. 12a. The study showed that maximum removal of TB dye and BPA was observed at 25 °C where, the TB and BPA removal percent decreased from 93.61 to 39.81% and 68.77 to 64.09% by FGS2 as the temperature increased from 25° to 50°, respectively. This shows that the TB and BPA adsorption processes were exothermic, implying that rising temperature reduces the forces of adsorption on the adsorbent surface between TB or BPA species and the active sites, lowering removal effectiveness^55^.

Thermodynamic studies provide vital information on internal energy variations, as well as the spontaneity and heat change of the adsorption process. The estimation of parameters standard free energy change (ΔG°), the standard enthalpy change (ΔH°), and the standard entropy change (ΔS°) is presented in S-6. ΔH°, ΔS° and ΔG° values are calculated and tabulated in Table 4. The exothermic nature of the adsorption mechanism was suggested by the negative predicted values of ΔH°. These results were proportional to the percentage of TB or BPA removed by FGS2 decreasing as the temperature of the aqueous solutions increased. During TB and BPA adsorption, the negative values of ΔS° suggest a decrease in disorder at the solid/liquid interface. This is explained by the fact that as the temperature rises, the ion mobility of TB and BPA rises, leading the ions to escape from the solid to the liquid phase. As a result, the amount of aromatic that could be adsorbed was reduced. The negative G° indicated that the adsorption was a spontaneous process, and the value of ΔG° decreased as the temperature dropped, showing that lower temperatures enhanced TB and BPA adsorption on FGS2^56^.

**Table 4:** Thermodynamic parameters for TB and BPA adsorption on FGS2 composites.

Temperature (k)	Thermodynamic parameters		
	*ΔG°* (kJ/mol)	*ΔH°* (kJ/mol)	*ΔS°* (J/mol. K)
**TB**			
298	− 5.8354	− 94.8345	− 298.6547
303	− 4.3421		
313	− 1.3556		
323	1.6310		
**BPA**			
298	− 2.0104	− 7.4618	− 18.2933
303	− 1.9189		
313	− 1.7360		
323	− 1.5531		

### Adsorption mechanism

The adsorption mechanism was thoroughly investigated, revealing that TB dye and BPA had a strong affinity for FGS2. The structure and functional behavior of the adsorbate molecules, the surface of the adsorbent, and the interactions between the adsorbent and the adsorbate are all common factors that influence adsorption effectiveness. There are also other fundamental elements that influence aromatic molecule adsorption, such as surface area and the ability of the adsorbent's surface to interact with aromatic molecules.

The adsorbates in this study, TB and BPA, are aromatic chemicals with benzene rings. Because TB is a diazo and anionic dye, it is extremely soluble in water, whereas BPA is non-polar and just slightly so. GO/SiO_2_ hybrid nanocomposite, on the other hand, comprises a benzene ring-like structure with multiple oxygen-containing groups on its edges and basal plane on the graphene component, as well as oxygen-containing groups in silica. It is possible to adsorb TB and BPA via π–π attractive forces, hydrophobic forces, and hydrogen bonding interactions, depending on the structure of the adsorbate and adsorbent. Furthermore, the surface of GO/SiO_2_ has a cavity structure consisting of a hydrophobic inner cavity and a hydrophilic outer surface, and through hydrophobic forces, van der Waals forces, and other mechanisms, it may form relatively stable inclusion complexes with TB and BPA molecules. As a result, the adsorption mechanism for FGS2 could be synergistic adsorption combining several adsorption forces, with chemical adsorption dominating and physical adsorption serving as a complement.

**Figure 13:**
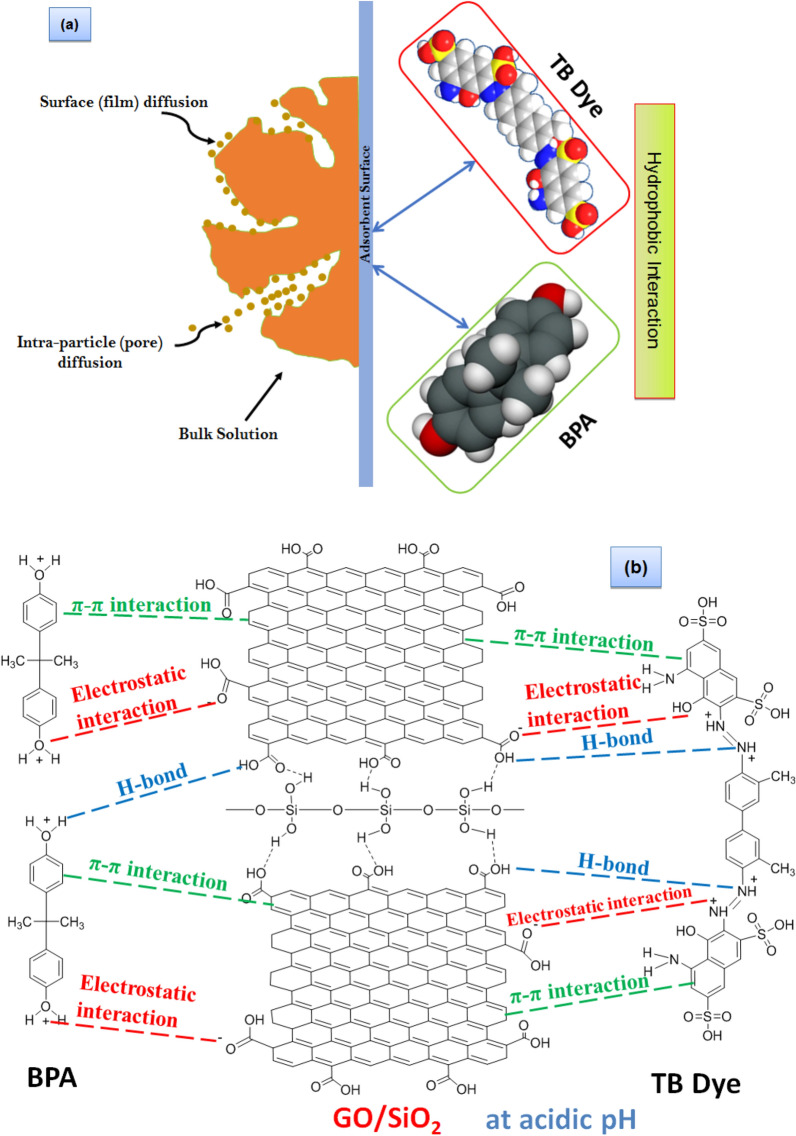
(**a**) Pore filling and hydrophobic interaction; and (**b**) Electrostatic interaction, π-π interaction, and H-bonding mechanism for removal of TB dye and BPA onto GO/SiO_2_ hybrid nanocomposite.

Between the hydroxyl groups of BPA or TB and the hydrophilic groups (such as carboxyl groups and hydroxyl groups) of FGS2, there are two types of adsorbate-adsorbent interactions: hydrogen bonding and π–π interaction between the benzene ring in BPA and the phenyl groups in FGS2. The adsorption performance of organic pollutants on porous materials like GO/SiO_2_ was previously shown to be influenced not only by intrinsic characteristics (e.g., charge, size, and electronic states), but also by weak intermolecular interactions (e.g., hydrogen-bonding and – interactions), hydrophobic interactions, and electrostatic interactions^56,57^. The probable adsorption mechanisms involved in TB dye and BPA removal by FGS2 were shown in Fig. 13. The influence of the above factors on the adsorption of TB and BPA by GFS2 would be discussed in the following.

#### Surface area and pore filling

Firstly, adsorption was facilitated by the surface area and pore size of GO/SiO_2_, as well as the molecular dimension of TB and BPA (Fig. 13a). When there was no identifiable adsorption site, the adsorption capacity of adsorbents increased with the enlargement of the specific surface area^56^. As a result, FGS2 had the greatest surface area, making it more effective at removing TB and BPA. This showed that surface area was not the most important factor in adsorption, and that the adsorbent and adsorbates had a unique interacting mechanism. The FGS2 features a blend of micropore and mesopore structure, as seen in Fig. 7. So, TB and BPA enter the pores of FGS2 through pore filling.

#### Hydrophobic interaction

Secondly, because of the hydrophobic benzene ring skeleton of graphene and the hydrophobic features of BPS and BPA pollutants, the hydrophobic part of BPA or TB prefers to bond with the hydrophobic part of FGS2 (Fig. 13a). Bisphenol A, in particular, has a high hydrophobicity and can be coupled with the hydrophobic site on FGS2's surface. As a result, a hydrophobic interaction plays a role in FGS2's ability to absorb BPA^56^. However, the hydrophobic interaction is not the most important factor in TB and BPA adsorption on FGS2.

#### π—π electron donor–acceptor

Thirdly, the enhanced adsorption of aromatic compounds onto the GO/SiO_2_ was thought to be mostly due to – interactions^58^. GO possesses bonds and several hydroxyl groups on its surface, as previously stated. As a result, it is better at adsorbing BPA and TB. The GO nanosheet's planner structure favors the presence of π–π. TB, as an organic dye, and BPA, as an electron acceptor, operate as electron acceptors in the residual -electron rich region of GO (Fig. 13b). As a result, we hypothesized that π–π interactions between the benzene rings of the GO/SiO2 and TB or BPA would increase dye and phenol adsorption, as previously described for graphene-based nanostructures^59,60^.

From another point of view, both TB and BPA have electrons that interact with the electrons of graphene's benzene rings via electron coupling, the interaction has traditionally been used to explain the mechanism of organic molecules with C=C double bonds or benzene rings adsorbed on the surface^60^.

#### Hydrogen bonding

Fourthly, because both TB and BPA have abundant hydrogen donors and acceptors, hydrogen bonding are anticipated to be a key component controlling their adsorption onto GO/SiO2^61^. The functional groups –OH and –C=O on the surface of FGS2 form a strong hydrogen connection with the functional groups –OH on BPA's structure and –O on TB's molecular structure (Fig. 13b).

#### Lewis acid–base (electrostatic interaction) interaction

Finally, in the adsorption process, the Lewis acid–base interaction played a key role. In acidic conditions, the adsorption capability of FGS2 to BPA or TB was determined. As a result, the protonation of the phenolic hydroxyl group in BPA, as well as the protonation of the azo (–N=N) group in the TB molecule, act as Lewis acids, resulting in Lewis acid–base interaction with the lone pair electrons on the oxygen-containing functional group (C–O) in GO. To put it another way, the phenolic hydroxyl groups in BPA and azo groups in TB are protonized, resulting in an electrostatic contact with the negatively charged GO/SiO_2_ surface (Fig. 13b).

**Figure 14:**
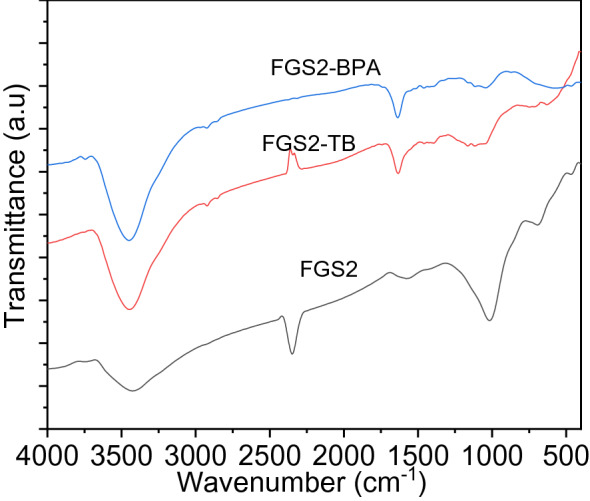
FTIR spectra before and after adsorption of BPA and TB onto FGS2.

The FTIR spectra of FGS2 before and after BPA and TB adsorption were compared to better understand the strengthening mechanism of GO/SiO_2_ towards TB and BPA. As shown in Fig. 14, the spectra collected after dye adsorption showed a large rise in the peak at 3446 cm^−1^, which can be attributed to the presence of hydrogen bonds and shows that such interactions may also be essential in the adsorption of organic dyes. Furthermore, the peak related to the stretching vibration of the C–O bond at 1013–1026 cm^−1^ shrank significantly. The skeletal vibration of aromatic C=C bonds was observed at 1633 cm^−1^ but sharper after adsorption, which was ascribed to the stretching vibration of C–H and the bending vibration of the aromatic rings, which finally verified the adsorption of TB and BPA on the FGS2 and also it had more potent π–π interactions. Furthermore, the stretching frequency of the O−H group had a small shift from 3455 to 3442 cm^−1^, which can be ascribed to hydrogen bonding between hydroxyl groups contained in both BPA and graphene^62^. These new peaks at 2800–3000 and 400–1750 cm^−1^ corresponded to peaks in the FTIR spectra of TB and BPA, and they occurred at significant intensities, indicating that a large number of TB and BPA molecules had been adsorbed on the graphene surface.

Unfortunately, deciphering the precise adsorption mechanism is a more difficult challenge that falls outside the scope of this study. However, the above findings imply that under acidic conditions, the tested anionic dye and bisphenol A can be easily adsorbed via electrostatic interactions and that these interactions play a substantial role in the adsorption of TB and BPA onto the surface of GO/SiO_2_. FTIR spectroscopy was fresh proof in proving the presence of the interaction and hydrogen bonding between BPA and graphene, based on the results of the above-mentioned investigations. Moreover, compared to other carbonaceous materials, graphene has larger and smoother surfaces for forming π−π interactions that can easily adsorb more organic contaminants.

### Extensive review on the performance of GO/ SiO_2_ adsorbent

To demonstrate the superiority of the GO/SiO_2_ adsorbent for removing TB or BPA from the aquatic environment, the results in Table S5 compare the adsorption capability of removing TB or BPA with other adsorbents. Table S5 shows that RHA-derived GO /SiO_2_ nanocomposites have a substantially higher affinity for TB dye and BPA than other nanocomposites described in the literature. In addition, Furthermore, RHA-derived GO/SiO_2_ nanocomposites have a substantially higher adsorption capacity and higher surface area than GO and/or its composites described in the literature^58–67^ and Table 5. Finally, the natural precursor of graphene nanocomposites, which were used in this study as low-cost adsorbents made by a chemical technique, provided an additional benefit.

**Table 5:** Comparable investigation onto the different types of graphene oxide materials.

Adsorbents	Adsorbate	Surface area (m^2^/g)	qm (mg/g)	Reference
rGO	Phenol	–	254.1	^63^
RGO	Phenol	1200	200	^64^
GO	Phenol	306	28.3	^65^
GO	Phenol	312	10.2	^66^
3D-GO	BPA	974.8	421	^67^
GO-CD	BPA	–	373	^68^
N-RGO	BPA	317	356	^67^
r-GO-CD	BPA	172	346	^69^
GO/HDTMA	BPA	–	141	^70^
T-rGO	BPA	494	87	^71^
H-rGO	BPA	139	70.4	^71^
RGO	Methylene blue	1200	53	^64^
N-RGO	Methylene blue	165	15	^72^
N-RGO	Methyl orange	165	38	^72^
N-RGO	Rhodamine B	165	5	^72^
GO/SiO2	TB	1768	455	Present study
GO/SiO2	BPA	1768	500	Present study

## Conclusions

In conclusion, we have demonstrated the synthesis of functionalized GO/SiO_2_ hybrid nanocomposites from a natural and sustainable precursor, rice husk, in a simple, scalable, and cost-effective manner. XRD, EDX, FTIR, Raman, SEM, HR-TEM, and BET surface area were used to completely characterize the hybrid composites. It proved that silica was decorated in graphene oxide. The functional groups of silane, hydroxyl, carboxyl, ketone, and epoxy were appropriately placed on the surface of graphene oxide sheets, as could be seen. FGS2 hybrid nanocomposite has a BET surface area of 1768 m^2^/g. FGS2's rapid, high, and selective adsorption of TB dye and BPA is attributed not only to its large surface area and microporous/mesoporous structure, but also to hydrogen bonding,—electron donor–acceptor, and electrostatic interaction in acidic environment. The primary parameters impacting the FGS2's strong adsorption capabilities are the π-π interaction and functional groups of the GO and silica. Kinetics, isotherm, and thermodynamic investigations, as well as the characterization of FGS2, were used to confirm the mechanism of TB dye and BPA adsorption onto GO/SiO_2_ hybrid nanocomposites. The adsorption isotherms were well-fitting to the Langmuir model, indicating monolayer adsorption, and the kinetics of adsorption was pseudo-second-order. FGS2 has a maximum adsorption capacity of 455 mg/g for TB and 500 mg/g for BPA, respectively. The free energy rose as the solution temperature decreased, indicating that the overall adsorption process was exothermic, according to the results of thermodynamic experiments. Overall, the synthesis strategy revealed that RHA could be converted into high-value-added materials, and it creates a new platform for the synthesis of GO/SiO_2_ hybrid nanocomposites in a rapid, reliable, efficient, scalable, and cost-effective manner, with FGS2 having remarkable sorption capability.

## Supplementary Information

Supplementary Information.

## Data Availability

The generated and analyzed data during the current study is supplied in this manuscript and is readily available from the corresponding authors upon reasonable request.
