# Supplementary material for: Controlled synthesis of graphene oxide/silica hybrid nanocomposites for removal of aromatic pollutants in water
Source: Sci Rep. 2022 Apr 29;12:7060. doi: 10.1038/s41598-022-10602-4 (PMC9054805; doi:10.1038/s41598-022-10602-4)
Supplement: Supplementary file 1 — Supplementary Information. [file 41598_2022_10602_MOESM1_ESM.docx]

**Supplementary Materials**

**Controlled synthesis of graphene oxide/silica hybrid nanocomposites for removal of aromatic pollutants in water**

Amr Abdelkhalek^1^, Mona Abd El-Latif ^2^, Hesham Ibrahim^1^, Hesham Hamad ^2,*^, Marwa Showman^2,*^,

^1^ Department of Environmental Studies, Institute of Graduate Studies and Research (IGSR), Alexandria University, P.O. Box 832, Alexandria, Egypt.

^2^ Fabrication Technology Research Department, Advanced Technology and New Materials Research Institute (ATNMRI), City of Scientific Research and Technological Applications (SRTA-City), New Borg El-Arab City, Alexandria 21934, Egypt.

**Corresponding author Email**: [heshamaterials@hotmail.com](mailto:heshamaterials@hotmail.com), [hhamad@srtacity.sci.eg](mailto:hhamad@chem.uw.edu.pl) (Hesham Hamad), [marwashowman@yahoo.com](mailto:marwashowman@yahoo.com) (Marwa Showman).

***S-1. Characterization techniques***

The functional groups of the prepared nanocomposites and ashes was recorded by using Fourier transform infra-red (FTIR 8400 S Shimadzu, Japan) in the spectrum range from 400 to 4000 cm^-1^ with scanning rate 2 cm^-1^ s^-1^. Raman spectroscopy was obtained for nanocomposites by using (SENTERRA, Bruker Optics, USA), The Raman spectra obtained from 500 cm^-1^ to 4000 cm^-1^ at room temperature. The compositional analysis and elemental ratios of the prepared nanocomposites and ashes were carried out by Energy Dispersive X-Ray (EDX) analysis combined with the scanning electron microscopy SEM (JEOL JSM 6360LA, Japan). The crystallinity of the prepared nanocomposite was investigated by X-ray diffraction analyses (XRD, 7000 Shimadzu, Japan) at room temperature. The 2θ range for all samples was in the range from 5° to 80° with Scan speed of 4° per minute. Scanning electron microscope (SEM, JEOL JSM 6360LA, Japan) was used to investigate the surface morphologies of composites in addition to the precursors (RHAs), samples were prepared by gold coating to avoid the local electrical charges build up. Particle size of the prepared nanocomposites is performed using Transmission Electron Microscopy (JOEL JEM 100CX, Japan), the samples were prepared by sonication in ethanol and then a few drops of the resulting suspension were put onto a grid coated with carbon. The specific surface area of the nanocomposites was measured by Brunauer-Emmett-Teller (BET) technique based on nitrogen adsorption at 77 K by using (NOVA 1000, Quantachrome Instruments, USA).

***S-2. Adsorption studies using the prepared GO/SiO_2_ nanocomposites.***

The characteristic adsorption affinity of the prepared GO/SiO_2_ nanocomposites, GS1, GS2 , and GS3, was studied by two types of organic pollutants, one is anionic dye, Trypan blue (TB), and other is phenolic compound, Bisphenol A, using the batch technique.

Consequently, the variation at the different processing parameters through the treatment of pollutant, Trypan blue (TB) or Bisphenol A (BPA), was monitored by analyzing the effect of contact time over a time period 0-60 min’ time interval, doses of GO/SiO_2_ nanocomposites utilized changed in the range 0.1-0.4 g/ L, initial pollutant dye concentration of 50-150 mg/L, solution temperature over 25 to 50 °C and the pH that was varied in range 2-12. After completing the adsorption tests, the supernatant was separated from adsorbent by centrifugation at 4000 rpm for 10 min. The residual concentrations after adsorption were measured by UV/Vis. spectrophotometer at a wavelength 590 and 280 nm for TB dye and Bisphenol A, respectively. The removal percentage and adsorption capacity (q) in mg/g were calculated using the equations (1) and (2) respectively [7]:

$\boldsymbol{\% Removal = C}\text{o}\boldsymbol{- C/C}\text{o}$ (1)

$\boldsymbol{q = (C}\text{o}\boldsymbol{- C)V/m}$ (2)

Where $\boldsymbol{C}\text{o}$ and $\boldsymbol{C}$ are the initial and final pollutant concentrations respectively in mg/L, $\boldsymbol{V}$ is the volume of the solution given by (L) and $\boldsymbol{m}$ is the mass of adsorbent used (g).

The concentration of pollutant in the solution was measured using an ultraviolet–visible spectrophotometer (Spectro UV–Vis Double Beam PC Scanning, Model UVD-2960, Labomed, Inc.). All experiments were performed three times with three different adsorbents FGS1, FGS2, and FGS3 to compare between them and clarification of the best conditions of synthesis an effective graphene and silica nanocomposite for removal of TB and BPA.

***S-3. Kinetics, isotherms and thermodynamic adsorption behavior.***

To describe the adsorption mechanism, the adsorption kinetic studies were conducted using four kinetic models of pseudo-first-order (PFO), pseudo-second-order (PSO), Elovich, and intra-particle diffusion kinetic models. On the other hand, the adsorption equilibrium for pollutant adsorption onto the adsorbent was theoretically modeled using three different isotherm models of Langmuir, Freundlich, and Temkin equilibrium isotherm models. Adsorption thermodynamics of pollutant onto GO/SiO_2_ nanocomposites were conducted at the studied solution temperatures range (298-323 K).

***S-4. Estimation of the number of graphene layers.***

he estimation of the average number of graphene layers (n) in the treated graphite samples by two steps, firstly, the estimation of stacking heights, L_a_, was proposed by Debye-Scherer equation

L_a_ = 0.89 λ / β_002_ cos _002_ (3)

Where β is the full width half maxima-FWHM, and secondly detection of n by the following equation [34]

n = L_a_ /d_002_ (4)

Where d_002_ is the interlayer spacing were obtained by using data from XRD patterns.

***S-5. Estimation of separation factor (R_L_)***

The vital characteristic of the Langmuir isotherm could be exhibited by the dimensionless constant termed as equilibrium parameter or separation factor (*R_L_*) which is formulated as follow:

(5)

(5)

where *K_L_* and *C_o_* is the Langmuir constant and the initial pollutant concentration, respectively. Established R_L_ values point out the isotherm type which is irreversible (*R_L_* = 0), favorable (0 < *R_L_* < 1), linear (*R_L_*=1) or unfavorable (*R_L_* > 1).

***S-6. Estimation of thermodynamic parameters.***

The thermodynamic parameters are the standard free energy change (*ΔG°*), the standard enthalpy change (*ΔH°*), and the standard entropy change (*ΔS°*) for the adsorption of TB and BPA onto FGS2 nanocomposites at various temperatures were calculated and listed in Tables 4. The apparent thermodynamic parameters, ΔH^°^ and ΔS^°^, for TB and BPA adsorption using synthesized FGS2 are calculated from the slopes and intercepts of the linear variation of ln K_c_ vs. 1/T as shown in Fig. 11 (b) by using the following equation:

ln K_c_ = (ΔS^°^/ R) – (ΔH^°^/RT) (6)

Where R is the universal gas constant, 8.314 J mol^−1^ K^−1^ and T is the absolute temperature in Kelvin. The distribution coefficient K_c_ was calculated using the following equation:

K_c_ = $\frac{q_{e}}{C_{e}}$ (7)

The ΔG^°^ for the adsorption process is calculated by using the following equation:

ΔG^°^ = ΔH^°^ − TΔS^°^ (8)

**Table S1:** Kinetic and equilibrium isotherm models utilized to describe pollutant adsorption process onto the prepared GO/SiO_2_ nanocomposites.

| Kinetic Models | | | | | | |
| --- | --- | --- | --- | --- | --- | --- |
| Model | Linear form | | | Plot | | Parameter |
| Pseudo-first-order  (PFO) | ln (q_e_-q_t_) = ln qe –k_1_t | | | ln (q_e_ –q_t_) vs. t | | k_1_- pseudo-first-order adsorption rate constant (L/min)  q_t_-amount adsorbed (mg/g)  q_e_- amount of adsorption equilibrium (mg/g) |
| Pseudo-second-order  (PSO) | $\frac{t}{q}=\frac{1}{k_{2}q_{e}^{2}}+\frac{t}{q_{e}}$ | | | $\frac{t}{q}$vs. t | | k_2_ - equilibrium rate constant of pseudo-second order (g/ mg min)  q_e_ - amount of adsorption equilibrium (mg/g) |
| Intra-particle diffusion | $q_{t}=a+k_{\mathrm{int}}t^{0.5}$ | | | $q_{t}$vs. $t^{0.5}$ | | $k_{\mathrm{int}}$intra-particle diffusion rate constant (mg/g. min) |
| Boyd | B_t_ = −0.4977 − ln(1 − F) | | | B_t_ vs. t | | F is the fraction of solute adsorbed at any time, t (min), calculated from F = q_t_/q_e_ |
| Isotherm Models | | | | | | |
| Model | | Linear form | | | Plot | Parameter |
| Langmuir | | | $\frac{C_{e}}{q_{e}}=\frac{1}{q_{m}b}+\frac{1}{q_{m}}C_{e}$ | | $\frac{C_{e}}{q_{e}}$ Vs. C_e_ | q_m_ - Maximum adsorption  capacity (mg/g)  b- Langmuir isotherm constant |
| Freundlich | | | $\ln q_{e}=\frac{1}{n}\ln C_{e}+\ln K_{f}$ | | ln q_e_ vs. ln C_e_ | q_e_ – Equilibrium dye uptake on adsorbent (mg/g)  Ce- Equilibrium dye concentration (mg/L)  K_f_- Measure of adsorption capacity (mg/g)  n*_f_*- Adsorption intensity |
| Temkin | | | q_e_= B ln K_T_ + B lnCe  B = RT/b | | q_e_ vs. lnC_e_ | K_T_ – Temkin isotherm constant (L/g)  B – Temkin constant related to heat of sorption (J/mol)  R – Gas constant (8.314 J/mol K)  T – Absolute temperature (K) |

**Table S2.** The pseudo-first order (PFO) and pseudo-second-order kinetic (PSO) parameters for TB and BPA removal using FGS2 adsorbent

| Kinetic model | |  | PFO | | |  | PSO | | |
| --- | --- | --- | --- | --- | --- | --- | --- | --- | --- |
| Initial pollutant concentration  (mg/L) | *q_exp_*  (mg/g) |  | *k_1_*  (min^−1^) | *q_e_*  (mg/g) | R^2^ |  | *k_2_*  (g/mg.min) | *q_e_*  (mg/g) | R^2^ |
|  |  |  |  |  |  |  |  |  |  |
| **TB** | | | | | | | | | |
| 50 | 205.82 |  | 0.0921 | 99.570 | 0.9933 |  | 0.4381 | 217.39 | 0.9990 |
| 75 | 269.70 |  | 0.0866 | 107.91 | 0.9891 |  | 0.4545 | 285.71 | 0.9993 |
| 100 | 375.82 |  | 0.1088 | 206.53 | 0.9993 |  | 0.4098 | 400.00 | 0.9996 |
| 150 | 430.15 |  | 0.1018 | 182.29 | 0.9956 |  | 0.4286 | 476.19 | 0.9994 |
| **BPA** | | | | | | | | | |
| 50 | 233.03 |  | 0.0984 | 201.95 | 0.9979 |  | 0.1162 | 303.03 | 0.9975 |
| 75 | 332.13 |  | 0.1025 | 257.88 | 0.9956 |  | 0.1689 | 400.00 | 0.9980 |
| 100 | 414.48 |  | 0.1251 | 270.02 | 0.9952 |  | 0.2973 | 454.55 | 0.9997 |
| 150 | 434.39 |  | 0.1317 | 230.30 | 0.9972 |  | 0.4565 | 476.19 | 0.9999 |

**Table S3:** Langmuir, Freundlich and Temkin isotherms equilibrium parameters for TB and BPA adsorption using FGS2 nanocomposites.

| Equilibrium isotherm | | Langmuir constant | | | |  | | Freundlich constant | | | |  | | Temkin constant | | | | | |
| --- | --- | --- | --- | --- | --- | --- | --- | --- | --- | --- | --- | --- | --- | --- | --- | --- | --- | --- | --- |
| Pollutant |  | | *q_m_* (mg/g) | *b*  (L/mg) | R^2^ | |  | | *K_F_* (mg/g) | *n_f_*  (L/g) | R^2^ | | | | *K_T_* (L/mg) | | *B* (J/mol) | | R^2^ |
| TB | | | 454.55 | 0.4783 | 0.9950 | | | | 212.34 | 4.8924 | 0.8403 | | 27.8075 | | | 62.310 | | 0.8399 | |
| BPA | | | 500.00 | 0.1117 | 0.9889 | | | | 109.52 | 3.0084 | 0.8210 | | 0.8681 | | | 107.180 | | 0.8197 | |

**Table S4:** Dimensionless equilibrium parameter (*R_L_*) tested at different initial concentrations of TB and BPA onto FGS2.

| Initial concentration (mg/L) |  | *R_L_* | |
| --- | --- | --- | --- |
| Pollutant |  | TB | BPA |
| 50 |  | 0.0383 | 0.1483 |
| 75 |  | 0.0278 | 0.1127 |
| 100 |  | 0.0204 | 0.0803 |
| 150 |  | 0.0141 | 0.0555 |

**Table S5:**  Adsorption capacity of TB and BPA by graphene/SiO_2_ nanocomposites obtained from RHA in comparison to other literature values.

| Adsorbents | Adsorbate | Adsorption capacity , q_m_ (mg/g) | Reference |
| --- | --- | --- | --- |
| Zn modified Luffa sponge | TB | 47 | [1] |
| [nHAp@GO](mailto:nHAp@GO) | TB | 41 | [2] |
| PDDA/GO | TB | 50 | [3] |
| MgO nanoparticles | TB | 132 | [4] |
| PAC-WS@AC | TB | 113 | [5] |
| Graphene/SiO_2_ from RHA | TB | 455 | Present Study |
| Rice husk ash | BPA | 9 | [6] |
| Graphene | BPA | 182 | [7] |
| [AgBr@rGo](mailto:AgBr@rGo) | BPA | 80 | [8] |
| Activated carbon from rice straw | BPA | 182 | [9] |
| HCPNSs | BPA | 203 | [10] |
| Si@C | BPA | 116 | [10] |
| Magnetic grapefruit peel biochar | BPA | 342 | [11] |
| CuZnFe_2_O_4_–biochar composite | BPA | 263 | [12] |
| Activated charcoal | BPA | 229 | [13] |
| KOH-activated TPC | BPA | 123 | [14] |
| MAP-GBM | BPA | 324 | [15] |
| Vc-GBM | BPA | 133 | [16] |
| Magnetic carbon nanotube | BPA | 45 | [17] |
| Magnetic particles MP_DVB_ | BPA | 91 | [18] |
| SB-beta-CD | BPA | 121 | [19] |
| Biochar KLP | BPA | 220 | [20] |
| Fe_3_O_4_@Co/Ni-LDH | BPA | 239 | [21] |
| Graphene/SiO_2_ from RHA | BPA | 500 | Present Study |

MAP-GBM= magnesium ascorbyl phosphate-graphene-based monoliths.

Vc-GBM = three-dimensional-graphene-based monoliths.

MP_DVB_ = magnetic polydivinylbenzene.

powder activated carbon (PAC)

granular activated carbon (WS@AC)

Luffa sponge (LS)

graphene oxide-doped nano-hydroxyapatite ((nHAp@GO)


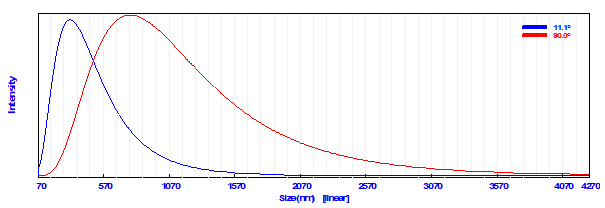


**(a)**


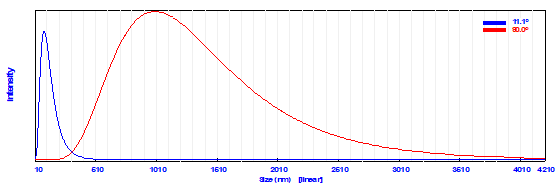


**(b)**

**Figure S1:** Particle size distribution of (a) RH, and (b) RHA.

| 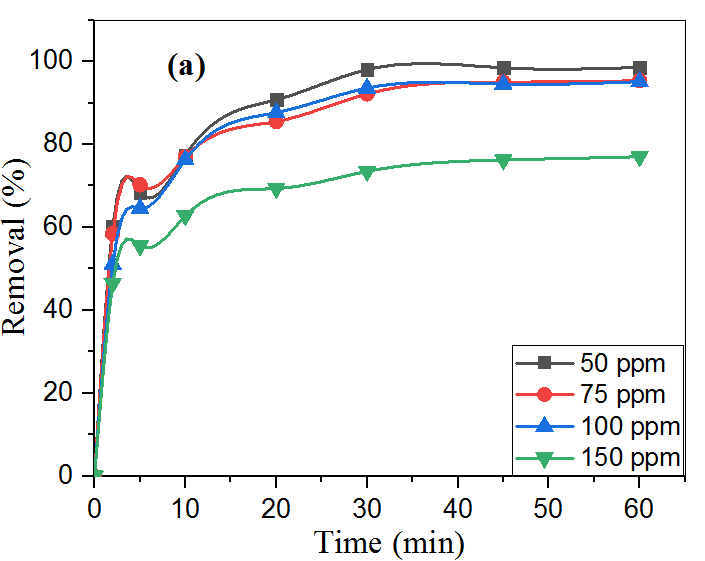 | 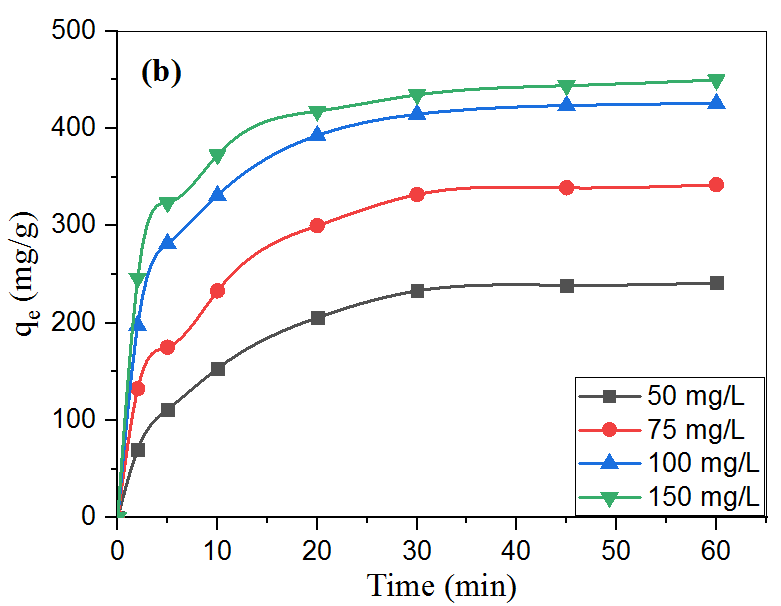 |
| --- | --- |
| 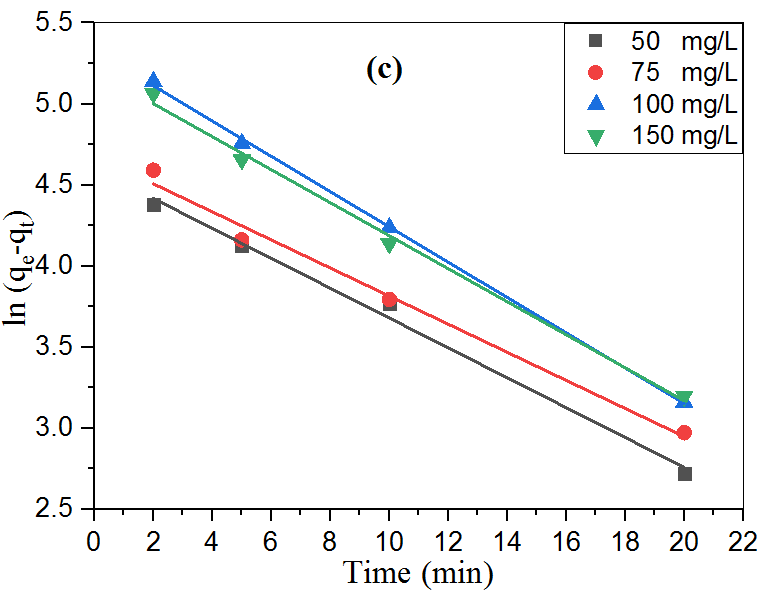 | 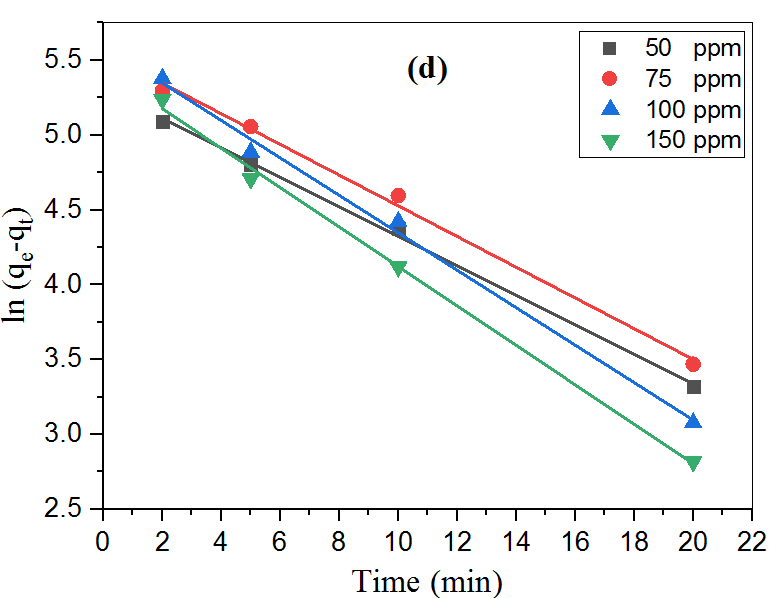 |
| 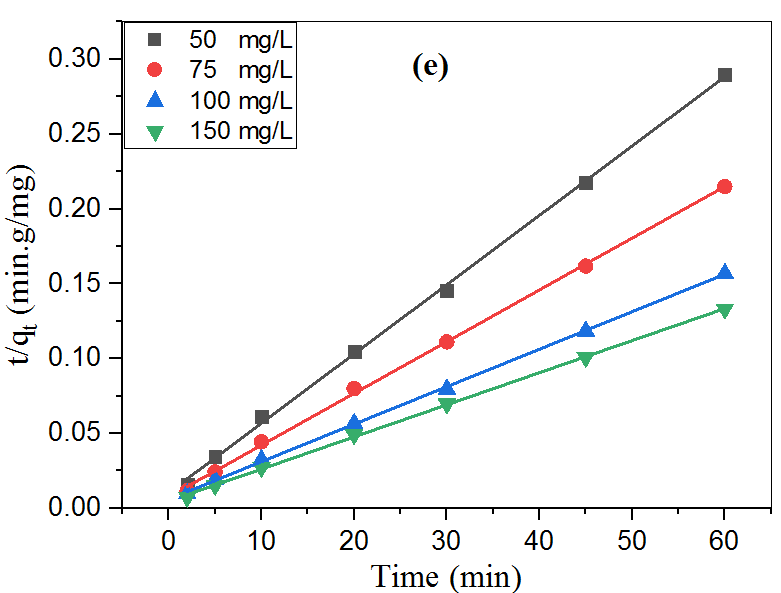 | 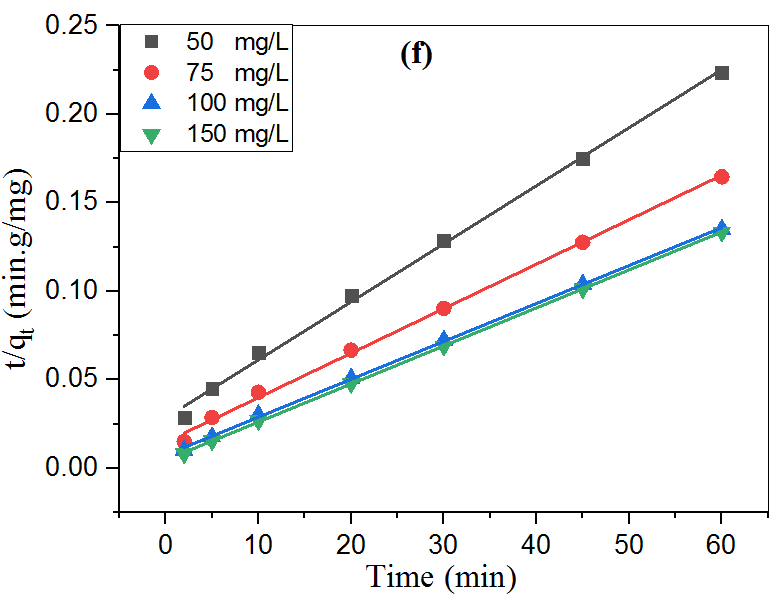 |

**Figure S2:** Effect of contact time and initial concentration of (a) TB and (b) BPA on the removal efficiency, Pseudo-first-order (PFO) kinetic plot of (c) TB, and (d) BP, and Pseudo-second-order (PSO) plot of (e) TB, and (f) BPA adsorption on FGS2 [pH: 3, agitation speed: 300 rpm, temperature: 25 °C and optimum adsorbent dose].

| 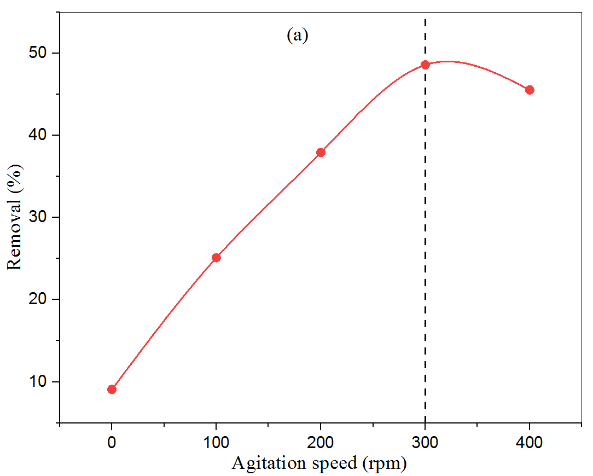 | 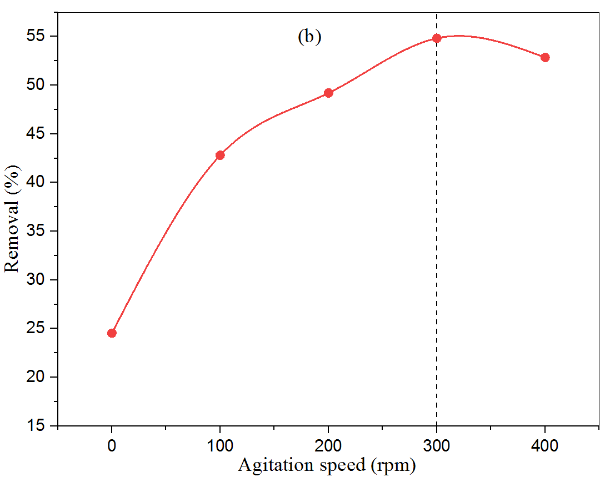 |
| --- | --- |

**Figure S3**: (a) Effect of agitation speed on adsorption of Trypan blue and (b) Effect of agitation speed on adsorption of BPA by FGS2 [initial concentration: 100 mg/L, adsorbent dose: 0.1 g/L, contact time: 30 min, pH: 3 and temperature: 25˚C].

| 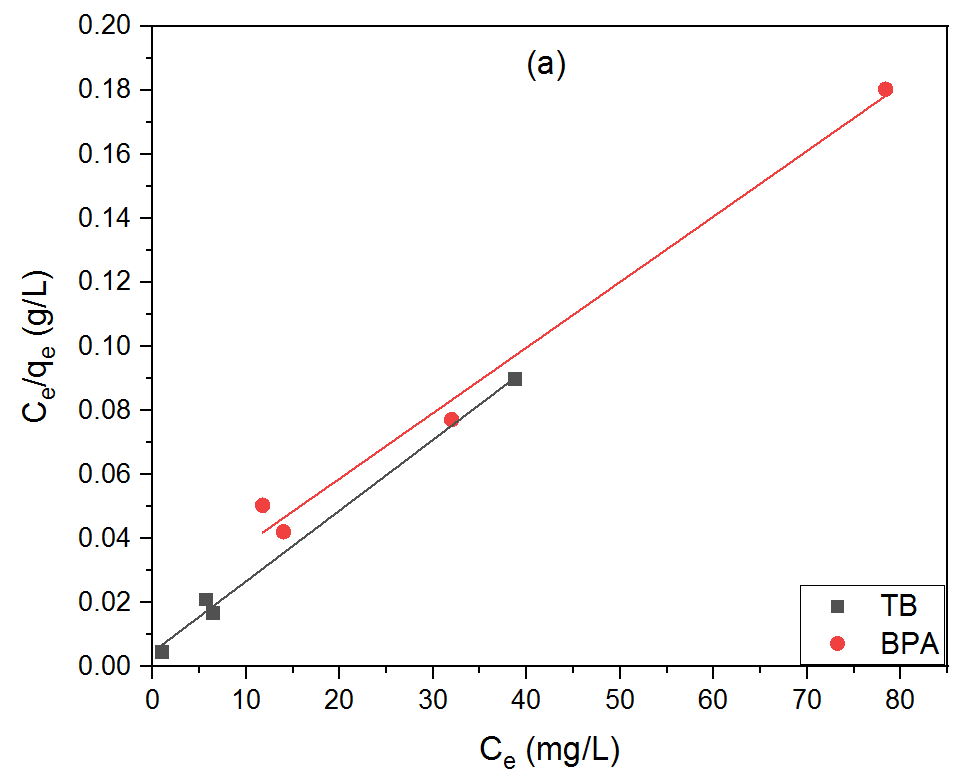 | 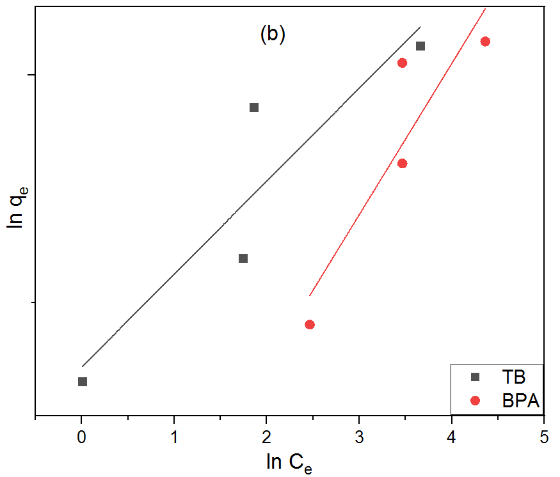 |
| --- | --- |
| 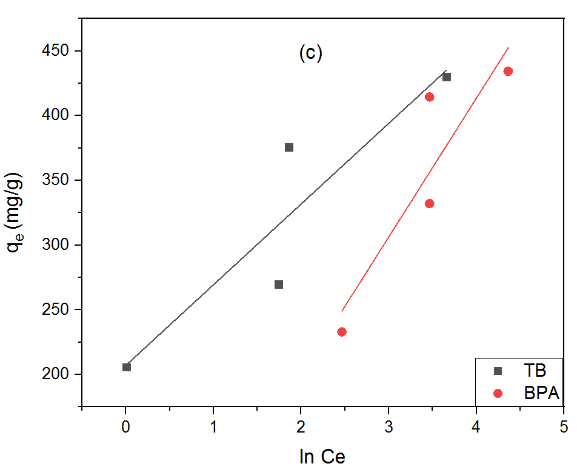 | |

**Figure S4: (a) Langmuir, (b) Freundlich, and (c) Temkin isotherm models plot for the adsorption of TB and BPA by FGS2 hybrid nanocomposites.**

References

1. H. Nadaroglu, S. Cicek, and A.A.Gungor, "Removing Trypan blue dye using nano Zn modified Luffa sponge," *Spectrochim. Acta A Mol.Biomol. vol. 172*, pp. 2-8, 2017.
2. S. M. Prabhu , A. Khan , M. H. Farzana , G. Ch. Hwang , W. Lee , and G. Le, "Synthesis and characterization of graphene oxide-doped nanohydroxyapatite and its adsorption performance of toxic diazo dyes from aqueous solution," *J. Mol. Liq.* vol. 269, pp.746–754, 2018.
3. X. Wang, Z. Liu, X. Ye, K. Hu, H. Zhong, J. Yu, J., . . . , and Z. Guo, "A facile one-step approach to functionalized graphene oxide-based hydrogels used as effective adsorbents toward anionic dyes," *App. Surf. Sci.* vol. 308, pp.82-90, 2014.
4. B. Priyadarshini, T. Patra, and T. R. Sahoo, "An efficient and comparative adsorption of Congo red and Trypan blue dyes on MgO nanoparticles: Kinetics, thermodynamics and isotherm studies," *J. Magnes. Alloy.*  vol. 9 , pp. 478–488, 2021.
5. Z. Cai, X. Deng, Q. Wang, J. Lai, H. Xie, Y. Chen, B. Huang, and G. Lin, "Core-shell granular activated carbon and its adsorption of trypan blue," *J. Clean. Prod.* vol. 242, 118496, 2020.
6. P. Sudhakar, I. D. Mall, and V.C. Srivastava, "Adsorptive removal of bisphenol-A by rice husk ash and granular activated carbon—A comparative study," *Des. Water Treat.* vol.57(26), pp. 12375-12384, 2016.
7. J. Xu, L. Wang, and Y. Zhu, "Decontamination of bisphenol a from aqueous solution by graphene adsorption," *Langmuir* vol. 28 (22) , pp. 8418-8425, 2012.
8. F. Chen, W. An, L. Liu, Y. Liang, and W. Cui, "Highly efficient removal of bisphenol A by a three-dimensional graphene hydrogel-AgBr@ rGO exhibiting adsorption/photocatalysis synergy," *App. Catal. B: Environ.* vol.217, pp.65-80, 2017.
9. K.-L. Chang, J.-F. Hsieh, B.-M. Ou, M.-H. Chang, W.-Y. Hseih, J.-H. Lin,… , and S.-T. Chen, "Adsorption studies on the removal of an endocrine-disrupting compound (Bisphenol A) using activated carbon from rice straw agricultural waste," *Sep. Sci. Technol.* vol. *47*(10), pp. 1514-1521, 2012.
10. P.K. Tripathi, L. Gan, M. Liu, X. Ma, Y. Zhao, D. Zhu,……, and N.N. Rao, "One-pot assembly of silica@ two polymeric shells for synthesis of hollow carbon porous nanospheres: adsorption of bisphenol A. *Mater. Lett.* vol.120, pp. 108-110, 2014.
11. J. Wang, and M. Zhang, "Adsorption characteristics and mechanism of bisphenol A by magnetic biochar," *Int. J. Environ. Res. Public Health* vol.17, pp.1075–1092, 2020.
12. J. Heo, Y. Yoon, G. Lee, Y. Kim, J. Han, and C.M. Park, "Enhanced adsorption of bisphenol A and sulfamethoxazole by a novel magnetic CuZnFe_2_O_4_–biochar composite," *Bioresour. Technol.* vol. 281, pp.179–187, 2019.
13. Y. Zhao, C.W. Cho, L. Cui, W. Wei, J. Cai, G. Wu, and Y.S. Yun, " Adsorptive removal of endocrine-disrupting compounds and pharmaceutical using activated charcoal from aqueous solution: equilibrium, kinetics, and mechanism studies," *Environ. Sci. Pollut. Res.* vol. 26, pp. 33897–33905, 2019.
14. R. Acosta, D. Nabarlatz, A. Sánchez-Sánchez, J. Jagiello, P. Gadonneix A. Celzard, and V. Fierro, "A0dsorption of bisphenol A on KOH-activated tyre pyrolysis char," *J. Environ. Chem. Eng.* vol. 6, pp. 823–833, 2018.
15. Z. Fang, Y. Hu, X. Wu, Y. Qin, J. Cheng, Y. Chen, P. Tan, and H. Li, "A novel magnesium ascorbyl phosphate graphene-based monolith and its superior adsorption capability for bisphenol A," *Chem. Eng. J.* vol. 334, pp.948–956, 2018.
16. Z. Fang, Y. Hu, W. Zhang, and R. Xian, "Shell-free three-dimensional graphene-based monoliths for the aqueous adsorption of organic pollutants," *Chem. Eng. J.* vol. 316, pp.24–32, 2017.
17. S. Li, Y. Gong, Y. Yang, C. He, L. Hu, L. Zhu, L. Sun, and D. Shu, "Recyclable CNTs/Fe_3_O_4_ magnetic nanocomposites as adsorbents to remove bisphenol A from water and their regeneration," *Chem. Eng. J.* vol. 260, pp. 231–239, 2015.
18. Z. Marzougui, A. Chaabouni, B. Elleuch, and A. Elaissari, "Removal of bisphenol A and some heavy metal ions by poly di vinyl benzene magnetic latex particles," *Environ. Sci. Pollut. Res.* vol. 23, pp.15807–15819, 2016.
19. F. M. Mpatani, A.A. Aryee, A. N. Kani, Q. Guo, E. Dovi, L. Qu, Z. Li, and R. Han, "Uptake of micropollutant-bisphenol A, methylene blue and neutral red onto a novel bagasse-â-cyclodextrin polymer by adsorption process," *Chemosphere* vol. 259, 127439,2020.
20. A. B. Hernández-Abreu, S. Álvarez-Torrellas, V.I. Águeda, M. Larriba, J. A. Delgado, P.A. Calvo, and J. García, "Enhanced removal of the endocrine disruptor compound bisphenol A by adsorption onto green-carbon materials. Effect of real effluents on the adsorption process,"  *J. Environ. Manag.*  vol. 266, 110604, 2020.
21. G. Li, X. Zhang, J. Sun, A. Zhang, and C. Liao, "Effective removal of bisphenols from aqueous solution with magnetic hierarchical rattle-like Co/Ni-based LDH," *J. Hazard. Mater.* vol. 381, 120985, 2020.
